# Ultrasonic repression of TRPA1-dependent astrocyte reactivity confers neuroprotection in models of Lewy body dementia

**DOI:** 10.1186/s40035-026-00544-6

**Published:** 2026-03-10

**Authors:** Ji Hun Kim, Keunhyung Lee, Minseok Koo, Doeun Kim, Jin Kyung Hong, Jeong-Yun Choi, Han Seok Ko, Joo-Ho Shin, Joo Min Park, Jinhyoung Park, Yunjong Lee

**Affiliations:** 1 Department of Pharmacology, Sungkyunkwan University School of Medicine, Samsung Biomedical Research Institute (SBRI), 300 Cheoncheon-Dong, Jangan-Gu, Suwon, Gyeonggi-Do 16419 Republic of Korea; 2 Department of Intelligent Precision Healthcare Convergence, Sungkyunkwan University, 300 Cheoncheon-Dong, Jangan-Gu, Suwon, Gyeonggi-Do 16419 Republic of Korea; 3 Neuroregeneration and Stem Cell Programs, Institute for Cell Engineering, Johns Hopkins University School of Medicine, Baltimore, MD 21205 USA; 4 Center for Memory and Glioscience, Institute for Basic Science (IBS), Daejeon, 34126 Republic of Korea; 5 University of Science and Technology (UST), Daejeon, 34113 Republic of Korea; 6 Department of Biomedical Engineering, Sungkyunkwan University, Suwon, Gyeonggi-Do 16419 Republic of Korea

**Keywords:** Lewy body dementia, Ultra-low-intensity ultrasound stimulation, Astrocyte, TRPA1, Inflammation

## Abstract

**Background:**

The pathology of Lewy body dementia (LBD) features neuronal α-synuclein (α-syn) accumulation and astrocytic hyperactivation in cognitive brain circuits. Ultra-low-intensity ultrasound (ULIUS) modulates astrocyte function via transient receptor potential ankyrin 1 (TRPA1) and has been investigated for therapeutic applications in neurodegenerative diseases.

**Methods:**

The therapeutic efficacy and mechanisms of ULIUS were evaluated in primary cultured astrocytes and neuron-glia cocultures treated with α-syn preformed fibrils (PFFs), as well as in an LBD model induced by hippocampal α-syn PFF injection into neuronal α-syn-A53T transgenic mice. Astrocytic TRPA1 was modulated under pathologic conditions with ULIUS or a pharmacologic TRPA1 antagonist to determine calcium responses and transcriptional regulation of *Trpa1* and inflammation-related genes. Neuropathological analyses for Lewy-like inclusions, neurodegeneration, and inflammation were performed in LBD mouse brains, with or without ULIUS. Spatial learning and memory were assessed using the Barnes maze.

**Results:**

Repeated transcranial ULIUS application was safe in long-term use and, unlike prolonged stronger ultrasound, did not cause hippocampal inflammation or neurodegeneration. It also prevented neuroinflammation and Lewy-like pathologies, rescuing cognitive impairment in LBD mice. ULIUS abolished α-syn-induced elevation of TRPA1, toll-like receptors-2 (TLR2), interleukin-1β, and tumor necrosis factor-α in LBD mouse brains. Mechanistically, both ULIUS and TRPA1 inhibitor blocked the sustained TRPA1-dependent calcium increase and the expression of inflammation-associated transcripts in α-syn PFF-treated astrocytes.

**Conclusions:**

Our findings provide mechanistic insights into the reciprocal TRPA1–TLR2 signaling pathway in α-syn-induced astrocyte pathology and underscore the disease-modifying potential of focused transcranial ULIUSm on astrocytes for the treatment of LBD. This study establishes a novel therapeutic strategy to alleviate neuroinflammation and cognitive decline associated with LBD. The demonstration of its long-term safety further supports ULIUS as a promising therapeutic strategy.

**Supplementary Information:**

The online version contains supplementary material available at 10.1186/s40035-026-00544-6.

## Introduction

α-Synucleinopathy is defined as the pathological accumulation of Lewy bodies and Lewy neurites mainly composed of misfolded α-synuclein (α-syn) aggregates [1–3]. The formation of neuronal α-syn aggregates disrupts the physiological function of neurons and causes progressive neuronal degeneration. Another pathologically important feature of α-syn aggregates is the transneuronal propagation and spread of Lewy pathologies to diverse brain structures, impairing corresponding behaviors during disease progression [4, 5]. Increasing evidence indicates that soluble α-syn oligomers, generated during the aggregation process or secreted after α-syn preformed fibrils (PFFs) seeding, are highly neurotoxic species. These oligomers impair synaptic function, disrupt membrane integrity, and contribute to early neuronal dysfunction [6]. In addition, α-syn oligomers strongly activate glial cells, particularly microglia and astrocytes, through pattern recognition receptors, leading to exaggerated inflammatory responses that amplify neuronal injury [7]. Thus, oligomers represent key mediators linking α-syn aggregation with both neuronal toxicity and glial activation. The formation or propagation of α-syn aggregates in the cortex and hippocampus can lead to impairment of synaptic circuits and cognitive impairment in Lewy body dementia (LBD), which includes Parkinson’s disease dementia and dementia with Lewy bodies [8, 9]. Indeed, α-syn PFF injection into mouse brains recapitulates the formation of Lewy-like inclusions and the propagation of α-syn pathology, mimicking α-synucleinopathy [5]. Extracellular α-syn PFF binds to lymphocyte-activation gene 3 for trans-neuronal transmission [10], whereas α-syn binding and activation of toll-like receptor 2 (TLR2) on glial cells promote inflammatory responses, which then potentiate neuronal toxicity [11]. Although LBD is the second most common neurodegenerative disease with cognitive impairments following Alzheimer’s disease, and patients suffer from a diverse spectrum of clinical symptoms, no effective disease-modifying treatments are currently available [2, 3, 12]. To be effective, it is critical for treatment strategies to target the pathological process at cellular and molecular levels underlying α-syn pathology propagation and glial inflammation in specific brain structures affected by LBD.

Low-intensity focused ultrasound stimulation (LIFUS) has received considerable attention for activating brain cells via mechanosensitive ion channels such as piezo and transient receptor potential (TRP) channels [13–16]. Neuromodulation by LIFUS has been studied in neurodegenerative diseases. In patients with Parkinson’s disease (PD), motor functions can be improved by LIFUS on the subthalamic nucleus where electrical deep brain stimulation is applied [17, 18]. Furthermore, LIFUS ameliorated cognitive decline in α-syn transgenic mice [19]. LIFUS may modulate astrocytic functions, which can help repair degenerated neurons or facilitate neuroplasticity through generation of new synaptic junctions, even in the presence of toxic protein aggregates of α-syn and amyloid-β [20, 21]. Furthermore, LIFUS can reduce inflammatory responses in animal models of PD [22, 23]. As deep brain stimulation can lower inflammatory responses by suppressing glial activation and pro-inflammatory cytokine production [24], LIFUS may have similar cellular mechanisms for its anti-inflammatory therapeutic effects. Despite the observed improvements in motor/cognitive brain functions, reduction of toxic α-syn aggregates, and modulation of glial inflammatory responses, astrocytic activity-related mechanisms underlying the therapeutic outcomes of ultrasound treatment in animal models of LBD and PD remain unknown.

While LIFUS at an intensity > 3 W/cm^2^ can directly activate both neurons and glial cells [16, 25], ultra-low-intensity (< 1 W/cm^2^) ultrasound stimulation (ULIUS) of brain tissue under physiological conditions can induce astrocyte-specific excitation via the TRPA1 calcium channel without directly activating healthy neurons, as the mechanosensitive TRPA1 ion channel has higher expression in astrocytes than in neurons [26]. Additionally, TRPA1 is an important mediator of inflammatory responses in glial and neuronal cells [26, 27]. TRPA1 transcription is increased in the brains of a 6-hydroxydopamine PD mouse model [28], and potential mechanisms underlying TRPA1 induction have been identified as being mediated by inflammatory cytokines like tumor necrosis factor (TNF)-α and interleukin (IL)-1β, in non-neuronal cells [29]. Considering the importance of astrocytic activation in neuroinflammation and the mechanosensitive characteristics of TRPA1 channels, TRPA1 may be a therapeutic target of ultrasound stimulation for treatment of PD. In PD, intracellular or extracellular accumulation of α-syn aggregates leads to switch of healthy astrocytes into the reactive state, thereby increasing inflammatory responses [30]. Pharmacological inhibition of TRPA1 suppresses inflammation and neurotoxicity in several animal models of neurodegenerative diseases [27, 31]. However, it remains unknown whether ULIUS can modulate TRPA1 activity and expression under PD-relevant pathological conditions, particularly in the presence of α-syn aggregation and propagation. Although studies have reported calcium changes in healthy neuronal cells in response to ULIUS [14, 32], little is known regarding the impact of astrocyte-selective ULIUS on the cellular, molecular, and calcium profiles of inflammatory brain cells.

Given the prominent roles of α-syn accumulation and glial hyperactivation in neurodegenerative diseases, therapeutic strategies that modulate astrocyte-driven neuroinflammation may be beneficial. Previous studies have shown that astrocytic TRPA1 contributes to calcium signaling and inflammatory responses, and that ULIUS can noninvasively modulate astrocyte function. However, whether ULIUS can therapeutically influence astrocytic TRPA1 signaling under α-syn–associated pathological conditions and thereby mitigate neuroinflammation and disease progression in LBD remains unknown. In this study, we investigated the therapeutic potential and underlying mechanisms of ULIUS in cellular and mouse models of LBD, with a particular focus on TRPA1-regulated astrocyte-mediated inflammatory pathways. To this end, we employed α-syn PFF–based in vitro systems and a hippocampus-targeted LBD mouse model to assess the effects of ULIUS on astrocytic signaling, neuroinflammation, neuropathology, and cognitive function.

## Materials and methods

### Purification of recombinant α-syn and preparation of α-syn PFFs

The pRK172-human-α-syn plasmid was transformed into BL21 (DE3)-RIL-competent *Escherichia coli* (Agilent, Santa Clara, CA; catalog no. 230245). The transformed BL21 (DE3)-RIL cells were cultured overnight at 37 °C to facilitate large-scale protein production. The cells were then lysed by lysis buffer containing 500 mM NaCl, 0.5% NP40 (Sigma-Aldrich, St. Louis, MO; catalog no. NP40S), 50 mM Tris, 5 mM ethylenediaminetetraacetic acid (EDTA), 5 mM ethylene glycol tetraacetic acid, 0.1% 2-mercaptoethanol (BIO-RAD, Hercules, CA; catalog no. 1610710), 1 mM phenylmethylsulfonyl fluoride (Sigma-Aldrich, St. Louis, MO; catalog no. PMSF-RO) in distilled water (DW), followed by sonication (Sonics, Newtown, CT; VC750 [2 min, 2-s on/4-s off, Amp 30%]) to induce cell lysis. The cell lysate was boiled for 15 min in a 95 °C water bath and then cooled on ice. Subsequently, the cell lysate was centrifuged at 6000× *g* for 30 min. The lysate was dialyzed using a dialysis bag (Thermo Fisher Scientific, Waltham, MA; catalog no. 66110) to remove salts, filtered through a 0.22 μm filter (Sartorius, Goettingen, Germany; catalog no. S6534), and further purified using a HiTrap Q HP anion-exchange column (Cytiva, Uppsala, Sweden; catalog no. 17115404). Purified human α-syn was concentrated to 5 mg/mL using a centrifugal filter unit (Merck Millipore, Burlington, MA; catalog no. UFC 901024). The concentrated human α-syn was agitated at 1200 rpm for 7 days in a thermomixer (Thermomixer C; Eppendorf, Hamburg, Germany) to generate α-syn PFFs. The formed α-syn PFFs were sonicated (15 s, 1-s on/1-s off, Amp 20%) before use in experiments.

### Primary cortical neuron and glia co-culture

Brains from mouse embryos at gestational days 15–16 were carefully dissected to isolate the cortices. The cortices were then incubated in 0.05% trypsin–EDTA (TE), phenol red (Thermo Fisher Scientific; catalog no. 25300062) at 37 °C for approximately 15 min. After incubation, the 0.05% TE solution was completely removed. The cortices were resuspended in Neurobasal A medium (Thermo Fisher Scientific; catalog no. 10888022) and gently dissociated using a Pasteur pipette. This was followed by three washes with centrifugation at 1500 rpm for 3 min each to ensure thorough cleaning. The washed cells were resuspended in Neurobasal plating medium containing 2% B27 supplement (Thermo Fisher Scientific; catalog no. 17504044), 1 mM GlutaMax supplement (Thermo Fisher Scientific; catalog no. 35050061), 25 μM *L*-glutamic acid (Sigma-Aldrich, St. Louis, MO; catalog no. G1251), 10% horse serum (Thermo Fisher Scientific; catalog no. 26050088), 1% penicillin/streptomycin (P/S) (Thermo Fisher Scientific; catalog no. 15140122) in Neurobasal A media and filtered through a 40-μm cell strainer (SPL Life Sciences, Pocheon, South Korea; catalog no. 93040). The filtered cells were then plated onto plates pre-coated with poly-DL-ornithine hydrobromide (PDLO) (Sigma-Aldrich; catalog no. P0671) at appropriate cell densities. After 3 days, half of the medium was replaced with Neurobasal feeding medium containing 2% B27 supplement, 1 mM GlutaMax supplement, 1 mM 4-(2-hydroxyethyl)−1-piperazine ethanesulfonic acid (HEPES) solution (Sigma-Aldrich; catalog no. H3537), 10% horse serum, and 1% P/S in Neurobasal A media. From day 5 in vitro (DIV5), glial cells began to proliferate. On DIV7, the cells were ready for experimental use.

### Primary astrocyte culture

The cortices of P0–P2 neonatal mouse brains were carefully dissected, and the attached meninges were removed. The isolated cortices were finely chopped using a blade and transferred to a tube containing 0.25% TE (Thermo Fisher ScientificA; catalog no. 25200056). The tissue was then incubated at 37 °C for 30 min and vortexed every 10 min. Following incubation, the cells were centrifuged at 300×*g* for 5 min to pellet the cells, and the supernatant was removed. The cell pellet was resuspended in culture medium containing 10% fetal bovine serum (Thermo Fisher Scientific; catalog no. 26140079) and 1% P/S in Dulbecco’s Modified Eagle Medium (Thermo Fisher Scientific; catalog no. 11995073) and gently dissociated using a Pasteur pipette. The dissociated cells were filtered through a 40-μm cell strainer and plated into T75 flasks pre-coated with PDLO. Three days after plating, the medium was replaced and subsequently changed every 2 days until DIV7–8. To remove microglia, the cultures were shaken at 180 rpm for 30 min. The medium was then completely replaced with fresh medium, and the cultures were shaken at 240 rpm for 6 h to remove oligodendrocyte precursor cells. The cells were then washed several times, and incubated with 0.05% TE for 5–10 min to detach the astrocytes. The cells were centrifuged at 180×*g* for 5 min, and the resulting cell pellet was used for further experiments by seeding the appropriate cells.

### Cell conditioned media preparation and treatment

#### Astrocyte conditioned media (ACM)

Primary astrocytes were cultured for 3 days after seeding to allow for differentiation. Then, the medium was replaced with fresh medium, and the astrocytes were treated with 1 × PBS alone or with α-syn PFFs (final concentration, 5 μg/mL) diluted in 1 × PBS. Two hours later, the cells were subjected to ultrasonic treatment, which was repeated every 2 days for three sessions. Following the final ultrasound treatment, the medium was mixed at a ratio of 1:1 with fresh medium and applied to the primary neuron-glia co-culture for incubation for 7 days without changing the medium. The primary co-culture was cultured for an additional 7 days, with the medium being replaced every 2–3 days, and was subsequently harvested for analysis. 

#### Microglia conditioned media (MCM)

After seeding BV2 cells, they were treated the next day with 1 × PBS alone or α-syn PFFs (final concentration, 5 μg/mL) diluted in 1 × PBS. Forty-eight hours post-treatment, the medium was collected, mixed 1:1 with fresh medium, and applied to primary astrocytes. Primary astrocytes stimulated by MCM were subjected to ultrasound stimulation as described below. Six hours after the final ultrasound treatment, cells were harvested for analysis.

### In vitro ultrasound stimulation

Ultrasonocoverslip, an ultrasound transducer integrated with a glass coverslip proposed in a previous report [33], was used to activate cultured cells. During reverse-transcription polymerase chain reaction (RT-PCR), immunofluorescence, and western blot experiments, Ultrasonocoverslips were adhered to the surface of a 12-well plate using biocompatible vacuum grease (Dow Corning, Midland, MI; catalog no. VG150). When the cells were ready for use, we applied three trials of ultrasound stimulation with 2 min intervals in a single day, and this session was repeated every 2 days, for three times. The fundamental ultrasonic pulse sequence for the stimulation was 333-μs-long 6-MHz bursts with pulse-repetition frequency (PRF) of 1.5 kHz. Acoustic pressure was 0.11 MPa in negative pressure, with an intensity of 0.2 W/cm^2^ calculated as spatial-peak-temporal-average. These acoustic parameters were selected based on a previous report [33] that showed astrocyte-selective activation observed using calcium imaging with a neuron-astrocyte co-culture. In this experimental configuration, an acoustic absorber (Precision Acoustics, Dorchester, UK; catalog no. Aptflex F28) was integrated with the 12-well-plate cover to reduce acoustic reflections from the boundary of the transducer. Therefore, precise ultrasound stimulation delivery to the cultured cells was possible in a 12 well-plate setup.

### Ca^2+^ imaging and ultrasound stimulation

Calcium responses of cultured astrocytes were measured using an Olympus MVX10 Macro Zoom Fluorescence Microscopy. To visualize calcium influx into astrocytes, Fluo4-AM dye (Thermo Fisher Scientific; catalog no. F14201) was diluted with HEPES buffer to a concentration of 1 μM. Then 2 mL of the dye-diluted HEPES buffer was added to each well plate chamber containing astrocytes cultured on an Ultrasonocoverslip and incubated for 30 min. The HEPES buffer contained 150 mM NaCl, 10 mM HEPES, 3 mM KCl, 2 mM CaCl_2_, and 5.5 mM glucose (PH 7.3), with an osmolarity of 320 mOsmol/kg. While the calcium influx induced by ultrasound stimulation was monitored, the astrocyte-cultured Ultrasonocoverslip was attached to the surface of the petri dish (SPL Life Science, Pocheon, South Korea; catalog no. 10035) filled with PBS or α-syn PFFs (5 μg/mL). The same acoustic parameters were utilized in the calcium imaging experiment as that described in the previous 12-well plate experiment.

### Animal experiments

All animal experiments were approved by the Institutional Animal Care and Use Committee of Sungkyunkwan University (Approval number: SKKUIACUC2023-11–19-1) in compliance with the international guidelines for animal welfare. The mice were housed in an environment with a 12-h light–dark cycle and had ad libitum access to food and water. Tet-Off conditional α-syn (A53T) transgenic mice were generated by crossing TetP-α-syn (A53T) mice (Jackson Laboratory, Bar Harbor, ME; Stock # 012442) with CamkIIα-tTA mice (Jackson Laboratory; Stock # 024854).

### Mouse brain stereotaxic injection and ultrasonic stimulation

To induce LBD in 2-month-old α-syn (A53T) transgenic mice, 2 μL of α-syn PFFs (5 μg/μL) were stereotaxically injected into the hippocampus (anteroposterior, 2.0 mm from bregma; mediolateral, 1.3 mm; dorsoventral, − 2 mm), at a rate of 0.1 μL/min. Following completion of each injection, the needle was slowly withdrawn 10–15 min later. After 3 days of recovery, ultrasound stimulation was administered. To precisely target the ultrasound stimulation to α-syn PFF-treated area, a focused ultrasound transducer was anchored to the XYZ stereotactic stage. The transducer was then moved to the α-syn PFF injection site, which was marked on the surface of the mouse skull. Pulsed ultrasound of 1 MHz, 1.5 kHz PRF, 50 duty cycle, 500 ms total burst period, and 0.11 MPa acoustic pressure was delivered three times per day with 2-min intervals. This session was repeated every 3 days for 1 month. A total of 10 sessions were performed. All the stimulations were conducted on both hemispheres under respiratory anesthesia with 1% isoflurane. The body temperature of mice was maintained at 37 °C using a heating pad. The brains of mice were extracted for analysis 1 month after stereotaxic injection.

### Behavioral tests

#### Barnes maze

The Barnes maze was performed to test hippocampal-dependent spatial learning and memory. The Barnes maze consists of a circular 90-cm diameter platform, with 20 holes of 5 cm arranged at regular intervals along the perimeter. Through these holes, the mice can escape to a safe location. The test was conducted in an environment with strong lighting and sound stimuli. These aversive conditions motivated the mice to seek the safety hole, allowing them to utilize and recall spatial cues related to their surroundings, including sounds and light, to find the escape holes. During the experiment, each mouse was placed at the center of the platform and allowed to explore for a total of 300 s, and the time taken to locate and enter the escape hole was recorded. To assess both short- and long-term memory, Test #1 was conducted, followed by Test #2 after 1 day. Subsequently, Tests #3 and #4 were performed 3 and 8 days after Test #2, respectively. Behavioral data were analyzed using the SMART V3.0 software (Panlab, Barcelona, Spain; catalog no. 76–0681).

#### Y-maze test

To minimize potential reductions in locomotor activity due to anxiety, mice were acclimated to the experimental conditions prior to testing. The Y-maze apparatus consisted of three identical arms arranged at 120° angles. During the test, each mouse was placed at the end of a same arm and allowed to freely explore the maze for 5 min. Behavior was recorded using a video tracking system, and spontaneous alternation performance was analyzed. The alternation percentage was calculated as the ratio of successive entries into all three arms (triplets) to the total number of possible alternations.

#### Pole test

Mice were habituated to the pole apparatus in two preliminary sessions before testing. The apparatus was a 23-inch metal rod (9 mm in diameter) wrapped with bandage gauze to prevent slipping. For each trial, each mouse was placed at the top of the pole facing downward, with hind paws positioned so that the heels contacted the rod’s edge. The latency to descend to the base was recorded manually with a stopwatch. Each animal performed three trials, and the mean of the three latencies was used as the performance score.

#### Rotarod test

Mice were trained on the rotarod for two consecutive days before testing. On the third day, motor coordination and balance were assessed using an accelerating rotarod system (Panlab, Barcelona, Spain; catalog no. 76–0770). The rotation speed increased from 4 to 40 rpm over a 2-min period, and the latency to fall was automatically recorded. Each mouse completed three trials, and the average latency was used as the performance score. All tests were performed under consistent conditions, with the apparatus cleaned between animals to minimize olfactory cues.

### Immunoblotting

Cells were harvested using 0.05% TE. Frozen brain tissue samples were pulverized and homogenized to break down the cells. For the soluble fraction, lysis buffer I containing 10 mM Tris (pH 7.4), 150 mM NaCl, 5 mM EDTA, and 0.5% NP40 in DW was added to the homogenized tissue and cells for 30 min with vigorous vortexing every 5 min. An aliquot was then taken, and 0.5% sodium deoxycholate (NaDOC) (Sigma-Aldrich; catalog no. D6750) was added, followed by 30-min lysis to obtain the total fraction. The remaining lysates were centrifuged at 14,000 rpm for 10 min to pellet the debris. The supernatant containing the soluble fraction was collected, and the pellet was washed with 1 × PBS and resuspended in lysis buffer II containing 1% sodium dodecyl sulfate and 0.5% NaDOC in lysis buffer I. This was followed by a 30-min lysis and sonication (15 s, 1-s on/1-s off, Amp 20%). After sonication, the mixture was centrifuged at 14,000 rpm for 10 min, and the supernatant containing the insoluble fraction was collected. Protein concentrations of the lysates were determined using a Pierce bicinchoninic acid Protein Assay kit (Thermo Fisher Scientific; catalog no. 23227), and samples were prepared in sample buffer for western blot analysis. Primary antibodies against α-syn (BD Biosciences, San Jose, CA; catalog no. 610787, 1:5000), TLR2 (Abcam, Cambridge, UK; catalog no. ab209216, 1:5000) and TRPA1 (Novus Biologicals, Centennial, CO; catalog no. NB110-40763, 1:250) were used. Horseradish peroxidase (HRP)-conjugated mouse anti-β-actin (Sigma-Aldrich, St. Louis, MO; catalog no. A3854) was also used. HRP-conjugated goat anti-mouse IgG (Genetex, Irvine, CA; catalog no. GTX-213111-01, 1:5000) and goat anti-rabbit IgG (Genetex, Irvine, CA; catalog no. GTX-213110-01, 1:5000) were used as secondary antibodies. Bands were visualized using chemiluminescence, and band intensities were analyzed using the ImageJ software (NIH, http://rsb.info.nih.gov/ij/).

### Immunohistochemistry

The mouse brain tissue was sectioned at a thickness of 35 μm using a sliding microtome (Thermo Fisher Scientific, Waltham, MA; catalog no. HM430). The sections were placed on plates and incubated for 30 min with blocking buffer containing 0.2% TritonX-100 (Sigma-Aldrich, St. Louis, MO; catalog no. X100), 0.02% sodium azide (Glentham Life Sciences, Corsham, UK; catalog no. GE6920), and 10% normal goat serum (Thermo Fisher Scientific, Waltham, MA; catalog no. 01–6201) in 1 × PBS. Subsequently, the sections were incubated with primary antibodies overnight at 4 °C. The primary antibodies included rabbit anti-phospho S129 α-syn (Abcam; catalog no. ab51253, 1:1000), rabbit anti-ionized calcium-binding adaptor molecule 1 (Iba-1) (Wako, Osaka, Japan; catalog no. 019–19741, 1:1000), mouse anti-GFAP (Cell signaling Technology, Danvers, MA; catalog no. 3670, 1:1000), and rabbit anti-tyrosine hydroxylase (Novus Biologicals; catalog no. NB300-109, 1:1000). The sections were then washed and incubated with the appropriate biotinylated mouse secondary antibodies (Vector Laboratories, Newark, CA; catalog no. PK-6102) and rabbit antibodies (Vector Laboratories; catalog no. PK-6101) for 1 h. Following incubation, the tissues were washed multiple times and further processed for 30 min using an ABC kit solution. To visualize the target antibodies, 3,3-diaminobenzidine (Sigma-Aldrich; catalog no. D4293) was used as the HRP substrate. The tissues were mounted on microscope slides and subjected to Nissl staining. Finally, mounting was performed using the DPX slide mounting medium (Sigma-Aldrich; catalog no. 06522).

### Immunofluorescence

Cells and tissues fixed in 4% paraformaldehyde were treated with 0.1% Triton X-100 buffer for 10 min to enhance antibody penetration. Subsequently, they were blocked with blocking buffer (5% normal goat serum in 0.1% Triton X-100 buffer) for approximately 1 h. The samples were then incubated overnight at 4 °C with primary antibodies diluted in blocking buffer.

The primary antibodies used were rabbit phospho S129 α-syn antibody (1:1000), TLR2 antibody (1:500), and mouse microtubule-associated protein 2 (MAP2) (Sigma-Aldrich, St. Louis, MO; catalog no. M4403) (1:1000), mouse α-syn antibody (BD Biosciences; catalog no. 610787, 1:1000), mouse GFAP antibody (Cell signaling Technology; catalog no. 3670, 1:1000), and rabbit Iba-1 antibody (Wako; catalog no. 019–19741, 1:1000). After incubation, the samples were washed several times and treated with 1 × PBS containing fluorescent-labeled secondary antibodies. Alexa Fluor 568 donkey anti-rabbit IgG (Thermo Fisher Scientific; catalog no. A-10042), Alexa Fluor 488 goat anti-rabbit IgG (Thermo Fisher Scientific; catalog no. A-11008), Alexa Fluor 568 donkey anti-mouse IgG (Thermo Fisher Scientific; catalog no. A-10037), and Alexa Fluor 488 donkey anti-mouse IgG (Thermo Fisher Scientific; catalog no. A-21202) were used as secondary antibodies. After another round of washing, 4′,6-diamidino-2-phenylindole staining was performed. Finally, the samples were mounted on glass slides using immuno-mount (Thermo Fisher Scientific; catalog no. 10662815).

### Real-time PCR

RNA was extracted from cells and tissues lysed by QIAzol lysis reagent (QIAGEN, Hilden, Germany; catalog no. 79306), and 1 μg of complementary DNA (cDNA) was synthesized using the iScript cDNA synthesis kit (BIO-RAD; catalog no. BR1708891). Real-time PCR was performed using the QuantStudio 6 Flex Real-Time PCR System (Applied Biosystems, Foster City, CA) with SYBR Green PCR Master Mix (Applied Biosystems; catalog no. 4309155) according to the manufacturer’s instructions. The cycle threshold (Ct) values obtained were then quantified using the delta-delta-Ct (ΔΔCt) method. GAPDH was used as an internal loading control. The RT-qPCR primer sequences are listed in Supplementary Table S1.

### Statistical analysis

Quantitative results are expressed as mean ± standard error of the mean. Statistical significance was determined using unpaired two-tailed Student’s *t*-tests for comparisons between two groups and analysis of variance tests with Tukey’s honest significant difference post hoc analysis for comparisons involving more than three groups. Differences were considered statistically significant at *P* < 0.05. GraphPad Prism software (version 8.0) was used for data visualization and statistical analysis.

## Results

### Astrocyte modulation by ULIUS suppresses neuronal α-syn aggregation induced by α-syn PFF treatment

**Fig. 1:**
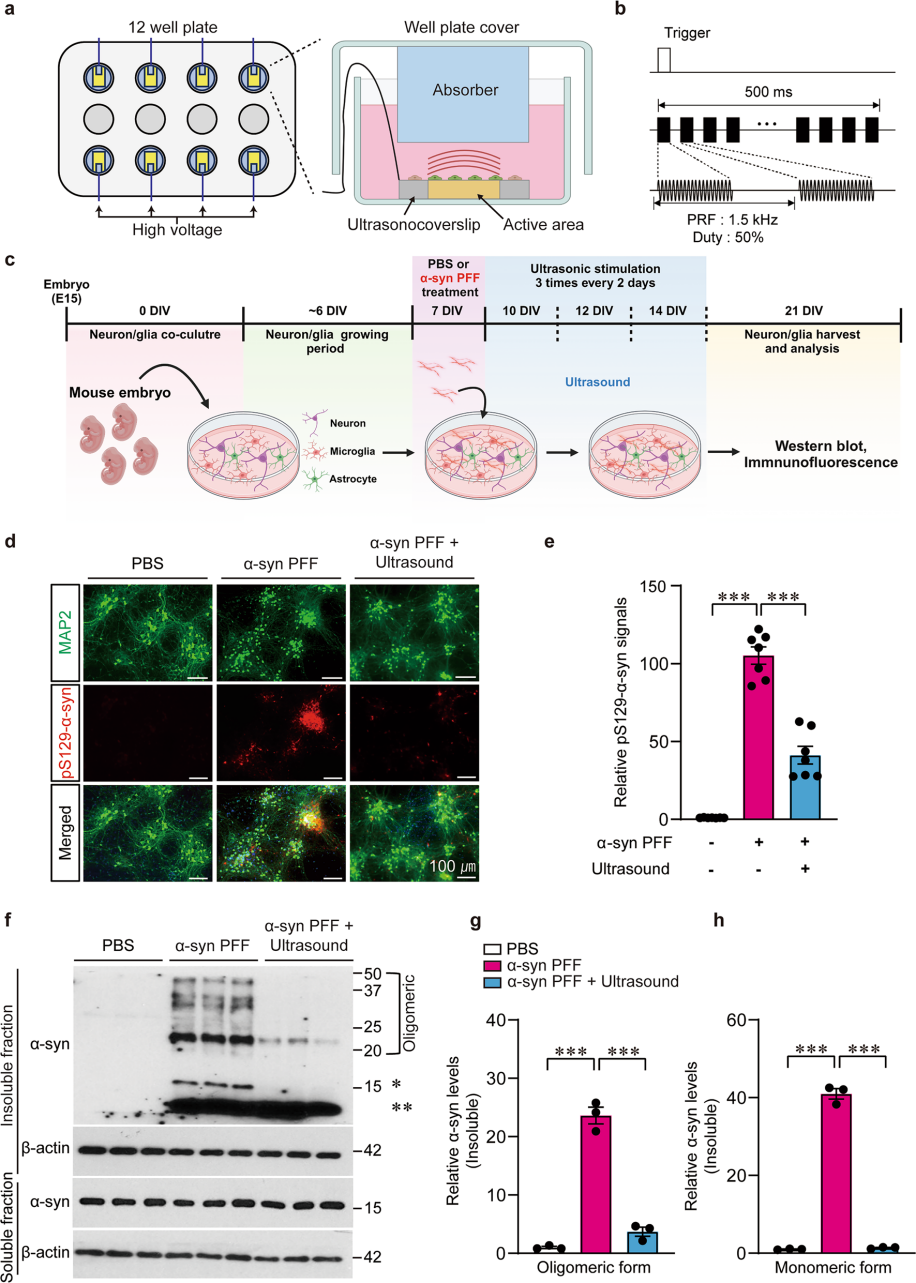
Ultrasound stimulations suppress α-syn aggregation in neurons treated with α-syn preformed fibril (PFF). **a** Schematic illustration of the ultrasound stimulation set-up and a cross-sectional view of Ultrasonocoverslip placed in the culture well plate integrated with an ultrasound absorber to suppress the reflections. **b** Acoustic parameters for activating astrocytes across the experiments. **c** Experimental scheme illustrating the procedures of α-syn PFF treatment and ultrasonic stimulations of primary cultured mouse cortical neurons and glia. **d** Representative immunofluorescence images of Lewy-like inclusion formations in primary cultured mouse cortical neurons treated with α-syn PFF (1 μg/mL) at 14 days after injection. Ultrasound was applied following the protocol in **b**. Scale bars, 100 μm. **e** Quantification of pS129-α-syn immunofluorescence signals (*n* = 7 per group). **f** Western blot analysis of α-syn aggregation in Triton X-100 insoluble protein fractions prepared from the primary cultured cortical neuron/glia with indicated PFF and/or ultrasound treatments. * indicates endogenous monomeric α-syn, whereas ** indicates exogenous α-syn PFFs. **g, h** Quantification of relative levels of monomer or oligomeric (high molecular weights) α-syn in the Triton X-100-insoluble fractions in each experimental sample (*n* = 3 per group). All data are presented as mean ± standard error of the mean. ***P < 0.001, one-way analysis of variance test followed by Tukey’s post hoc analysis

For *in-vitro* studies, we utilized an ultrasound transducer-loaded glass coverslip called ‘Ultrasonocoverslip’ [33]. As the diameter of the Ultrasonocoverslip was 15 mm, it could fit in each well of a 12-well culture plate, which was suitable for RT-PCR or western blot analysis (Fig. 1a). The Ultrasonocoverslip is composed of a glass coverslip, a piezoelectric layer of polyvinylidene fluoride trifluoroethylene, and a passivation layer of parylene (Fig. S1a). The operational frequency of the transducer was 6.1 MHz (Fig. S1b). The deviation of acoustic pressure field on the surface was within 1%, which can barely affect the variations in cellular responses (Fig. S1c, d). Furthermore, we placed an ultrasonic absorber plate on the cover of the 12-well plate, which was immersed in a buffer solution to minimize acoustic reflection between the transducer and the surface of the buffer solution (Fig. 1a). The acoustic reflection was significantly suppressed (up to 5.1 dB), using an acoustic absorber (Fig. S1e–g). In each sonication session, a 333-μs long, 6-MHz sinusoidal burst with a pressure of 0.11 MPa was repeatedly transmitted with a pulse-repetition-rate of 1.5 kHz for 500 ms, which enabled astrocyte-specific activation in wild-type neuronal cells (Fig. 1b) [33]. Primary brain cells were harvested from 15–16-day-old mouse embryo, and neurons, microglia, and astrocytes were co-cultured on the Ultrasonocoverslip. The cultured batches were separated into the following groups: control group receiving phosphate-buffered saline (PBS), sham group receiving α-syn PFFs to induce Lewy-like inclusion formation, and the experimental group receiving α-syn PFFs with ultrasound stimulation. In the experimental group, we applied three sessions of sonication with an interval of 2 min and repeated the stimulation training three times every other day (Fig. 1c). pS129-α-syn immunofluorescence revealed robust endogenous α-syn aggregation in MAP2-positive neurons at 14 days after α-syn PFF treatment (Fig. 1d, e). These α-syn PFF-induced pS129-α-syn-positive Lewy-like inclusions were largely repressed by ultrasound applications (Fig. 1d, e). Moreover, western blot analysis confirmed a marked elevation of detergent-insoluble α-syn aggregates in astrocyte-neuron co-cultures with α-syn PFF treatment, which was almost completely blocked by ULIUS with selective parameters for astrocytes (Fig. 1f–h). Together, these results show that ultrasound-mediated astrocyte stimulation was effective in suppressing the α-syn PFF-seeded neuronal α-syn aggregation.

### Inflammatory conversion of astrocytes with αSyn PFF treatment was blocked by ultrasound

**Fig. 2:**
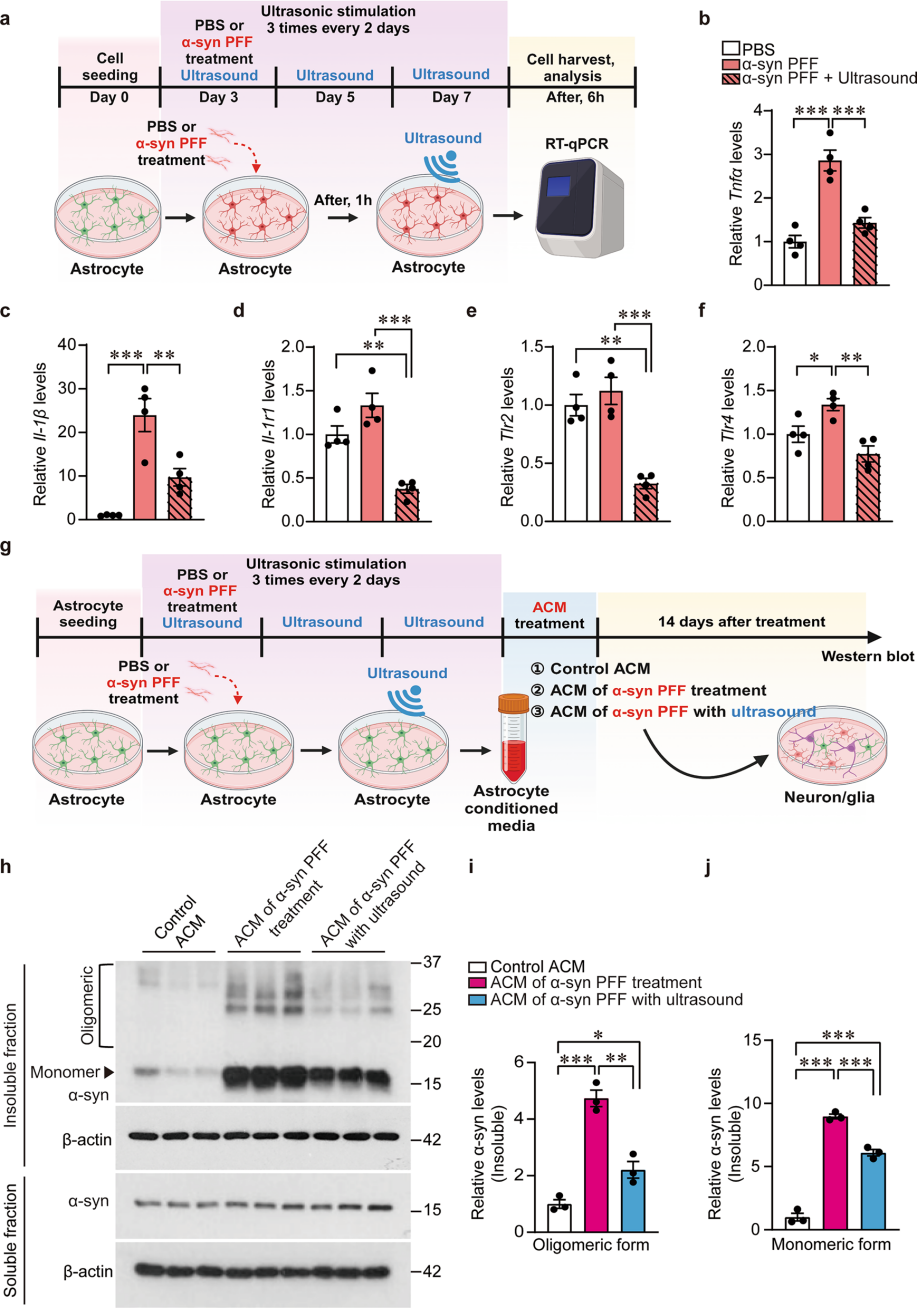
Ultrasound treatment to astrocytes inhibits α-syn preformed fibril (PFF)-induced astrocytic inflammatory responses and neuronal α-synucleinopathy. **a** Scheme of the experimental procedures for repeated ultrasound application to primary cultured astrocytes. **b–f** Expression of *Tnfα*, *Il-1β*, *Il-1r1*, *Tlr2*, and *Tlr4* determined by real-time qPCR (*n* = 4 per group). *Gapdh* was used as an internal loading control for normalization. **g** Scheme of experimental procedures for primary astrocyte culture, α-syn PFF treatment, and repeated ultrasound applications. Conditioned media prepared from astrocytes with the indicated treatments were subsequently added to primary cortical neuron/glia co-cultures to evaluate neuronal α-syn-related pathology. **h** Western blot analysis of α-syn aggregation in Triton X-100-insoluble protein fractions from primary cortical neurons cultured in the conditioned media prepared from primary astrocytes with the indicated treatments of PFF and ultrasound. β-Actin served as the internal loading control. **i, j** Quantification of monomer or oligomeric (high molecular weights) α-syn levels in the Triton X-100-insoluble fractions from each experimental sample (*n* = 3 per group). All data are presented as mean ± standard error of the mean. *P < 0.05, **P < 0.01 and ***P < 0.001, one-way analysis of variance test followed by Tukey’s post hoc analysis

Astrocytes respond to extracellular α-syn PFFs and are involved in neuronal α-syn pathologies under neuroinflammation [30, 34]. As astrocyte-selective ultrasound parameters were effective in modifying neuronal α-syn pathology (Fig. 1), we sought to determine the potential disease-modifying functions of astrocytes modulated by ultrasound. Mouse primary astrocytes were cultured on an Ultrasonocoverslip, and astrocytic PD models were induced by α-syn PFF treatment. Ultrasound was delivered three times every other day (Fig. 2a). Six hours after completion of the final sonication, the transcript levels of proinflammatory cytokines and receptor (*Tnf-α, Il-1β*, and *Il-1r1*) and α-syn PFF receptor on glia [35] (*Tlr2* and *Tlr4*) were measured. The transcript levels of proinflammatory cytokines TNFα and IL-1β, as well as TLR4, were increased in response to α-syn PFF treatment (Fig. 2b, c, f). Direct ultrasound application to astrocytes effectively prevented these increases. The expression of *Il-1r1* and *Tlr2*, which showed a trend of increase in α-syn PFF-treated astrocytes, was suppressed by ultrasound application to be lower than the PBS-treated control level, even in the presence of α-syn PFFs (Fig. 2d, e).

In addition to direct α-syn treatment, reciprocal interaction with microglia can induce conversion of astrocytes to the pathogenic reactive state in α-synucleinopathy. To evaluate whether ultrasound could prevent the reactive conversion of astrocytes, even under pathological interaction with inflammatory microglia [36, 37], we prepared BV2 cell MCM by treating BV2 cells with α-syn PFFs or PBS as a control (Fig. S2a). The conditioned media were added to primary astrocyte cultures, followed by ultrasound stimulation (Fig. S2a). RT-qPCR assessment showed robust increase in *Tnf-α* and *Il-1β* expression in astrocytes treated with the pathogenic MCM compared with that treated with control MCM (Fig. S2b, c). Similarly, the pathogenic MCM-induced increase in *Tnfα* and *Il-1β* expression was substantially reduced by ultrasound treatment of astrocytes (Fig. S2b, c). These findings indicate that ULIUS was effective in preventing the conversion of astrocytes into the pathological phenotype that expresses proinflammatory cytokines in response to either α-syn PFF binding or paracrine stimulation from reactive microglia.

Under physiological conditions, astrocytes play a supportive role in neuronal survival. However, under pathological conditions, reactive astrocytes exacerbate neuronal pathology including α-syn aggregation and propagation, via production of proinflammatory cytokines [35]. To determine the potential neurotoxicity conferred by astrocytes, we prepared ACM from astrocytes treated with PBS, α-syn PFFs, or α-syn PFFs with subsequent ultrasound applications (Fig. 2g). The ACM was then applied to mixed cultures of neurons and glia. After 2 weeks, the levels of α-syn aggregation was measured by western blotting (Fig. 2g). The pathologic ACM (media from α-syn PFF-treated astrocytes) led to a significant increase in neuronal α-syn monomer and aggregates in the detergent-insoluble fractions (Fig. 2h, i). The ACM of ultrasound-stimulated astrocytes reduced the levels of neuronal detergent-insoluble α-syn aggregates and monomers compared with the pathologic ACM-treated neurons (Fig. 2h–j). These results were consistent with the ultrasound suppression of astrocytic cytokine production (Fig. 2a–f) and confirm that the therapeutic effects of ultrasound on neuronal α-syn pathology occur through modulation of astrocytic function.

### Both ultrasound application and pharmacological TRPA1 inhibition repress αSyn PFF-induced TRPA1 expression, calcium influx, and inflammatory response in astrocytes

**Fig. 3:**
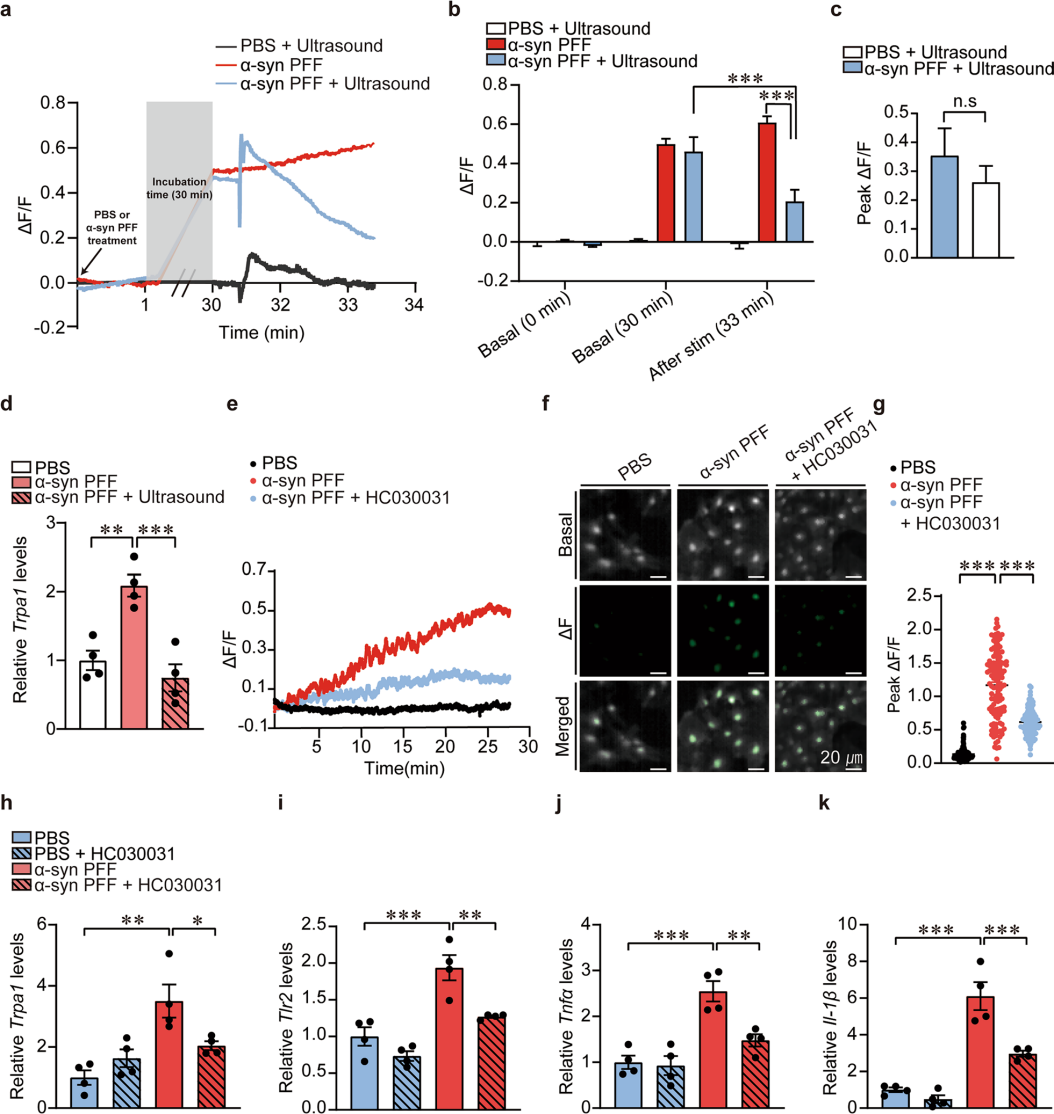
Ultrasound blocks the preformed fibril (PFF)-induced TRPA1 expression and sustained calcium influx in astrocytes, similar effects as pharmacological inhibition of astrocytic TRPA1. **a** Mean traces of calcium-sensitive fluorescence changes (ΔF/F_0_) of primary cultured mouse astrocytes at the basal level and 30 min after α-syn PFF/PBS treatment with/without ultrasound stimulation (*n* = 41, 72, 73 for PBS + ultrasound, PFF, and PFF + ultrasound groups, respectively). **b** Quantification of calcium changes in ΔF/F_0_ basal values at the first imaging frame, after 30 min of loading, and 2 min after ultrasound stimulation (*n* = 41, 72, 73 for PBS + ultrasound, PFF, PFF + ultrasound groups, respectively). **c** Peak calcium changes manifested by ΔF/F_0_ values (*n* = 73 and 72 for PFF + ultrasound and PBS + ultrasound groups, respectively). **d** Quantification of *Trpa1* mRNA expression in primary astrocytes treated with PBS or α-syn PFFs (5 μg/mL) along with ultrasonic stimulation (three times/day, 2 days a week), assessed by RT-PCR (*n* = 4 per group). **e** Average calcium traces in primary astrocytes treated with PBS or α-syn PFFs (5 μg/mL) alone or α-syn PFFs after application of HC030031 (40 μM, 20 min). **f** Representative fluorescence images of calcium in primary astrocytes. **g** Calculated maximum fluorescence intensity changes after treatment with PBS (*n* = 145), α-syn PFF (*n* = 127), and α-syn PFF + HC030031 (*n* = 105). **h–k** Real-time qPCR quantification of mRNA expression of *Trpa1*, *Tlr2*, *Tnfα*, and *Il-1β* in the primary astrocyte model of α-synucleinopathy induced by α-syn PFF treatment (5 μg/mL, 5 h), in the presence or absence of TRPA1 inhibition (HC030031-10 μM, 6 h). All data are presented as mean ± standard error of the mean. *P < 0.05, **P < 0.01 and ***P < 0.001, one-way analysis of variance test followed by Tukey’s post hoc analysis

Next, we sought to determine the molecular mechanisms underlying the astrocyte modulation by ultrasound. To understand whether reactive astrocytes were activated or suppressed by ultrasound, we measured the calcium responses of Fluo4-AM dye-loaded astrocytes cultured on Ultrasonocoverslip. Notably, α-syn PFF treatment of astrocytes led to a sustained and gradual increase in intracellular calcium levels (Fig. 3a, b). Consistent with a previous study [33], ultrasound treatment of PBS-treated control astrocytes resulted in a brief and sharp rise of calcium signal, which subsequently returned to baseline in 3 min (Fig. 3a). Notably, acute ultrasound application to α-syn PFF-treated astrocytes induced a brief rise in intracellular calcium followed by a steady decline, eventually leading to 66% suppression of calcium levels compared with the α-syn PFF-treated astrocytes (Fig. 3a, b). Interestingly, the relative extent of calcium increase immediately after ultrasound application was similar between the PFF- and the PBS-treated astrocytes (Fig. 3c).

To determine whether TRPA1 plays a critical role in neuroinflammation in LBD, we first monitored whether TRPA1 expression shows any pathological relevance. An approximately twofold increase in *Trpa1* mRNA levels in astrocytes was observed following α-syn PFF exposure for 7 days, and this increase was considerably blocked by repeated applications of ultrasound (Fig. 3d). To verify whether TRPA1 mediates the α-syn-induced sustained increase of intracellular calcium in astrocytes, the calcium levels were monitored in the presence/absence of the TRPA1 antagonist, HC030031. HC030031 robustly blocked this calcium increase in response to α-syn PFF treatment (Fig. 3e–g). As both ultrasound and TRPA1 inhibitor treatments prevented the α-syn PFF-induced astrocytic calcium influx, the inflammatory transcript profiles in the TRPA1 inhibitor-treated astrocytes were analyzed using RT-qPCR. Similar to the effects of ultrasound, TRPA1 inhibition by HC030031 repressed the α-syn PFF-induced expression of *Trpa1* and *Tlr2* (Fig. 3h, i). Moreover, the α-syn-induced expression of proinflammatory cytokines, *Tnfα* and *Il-1β*, was significantly suppressed by pharmacological blocking of TRPA1 (Fig. 3j, k). Inhibition of TRPA1 under basal condition did not alter the expression of *Trpa1, Tlr2*, and *Il-1β* compared to control (Fig. 3h–k).

We next examined whether ultrasound suppresses α-syn PFF-induced astrocytic inflammatory conversion through TRPA1 inhibition. To test this, we compared the effects of ultrasound, TRPA1 inhibitor, and their combined application on *Trpa1, Tlr2, Tnfα*, and *Il-1β* transcription in astrocytes following PFF treatment. The combined treatment did not produce greater suppression than either treatment alone, indicating that the ultrasound-mediated repression of astrocytic inflammation is largely achieved via TRPA1 inhibition (Fig. S2d–g). Taken together, these results indicate that TRPA1 inhibition induced similar therapeutic effects as ultrasound on the α-syn PFF-induced pathological alterations in astrocytes. The transcriptional upregulation of astrocytic *Trpa1* and *Tlr2* by α-syn PFFs suggests a potential feed-forward acceleration of pathologic conversion of astrocytes.

### Transcranial ultrasound prevents cognitive impairment and α-syn pathology formation and propagation in α-syn transgenic mice with hippocampal injection of α-syn PFFs

**Fig. 4:**
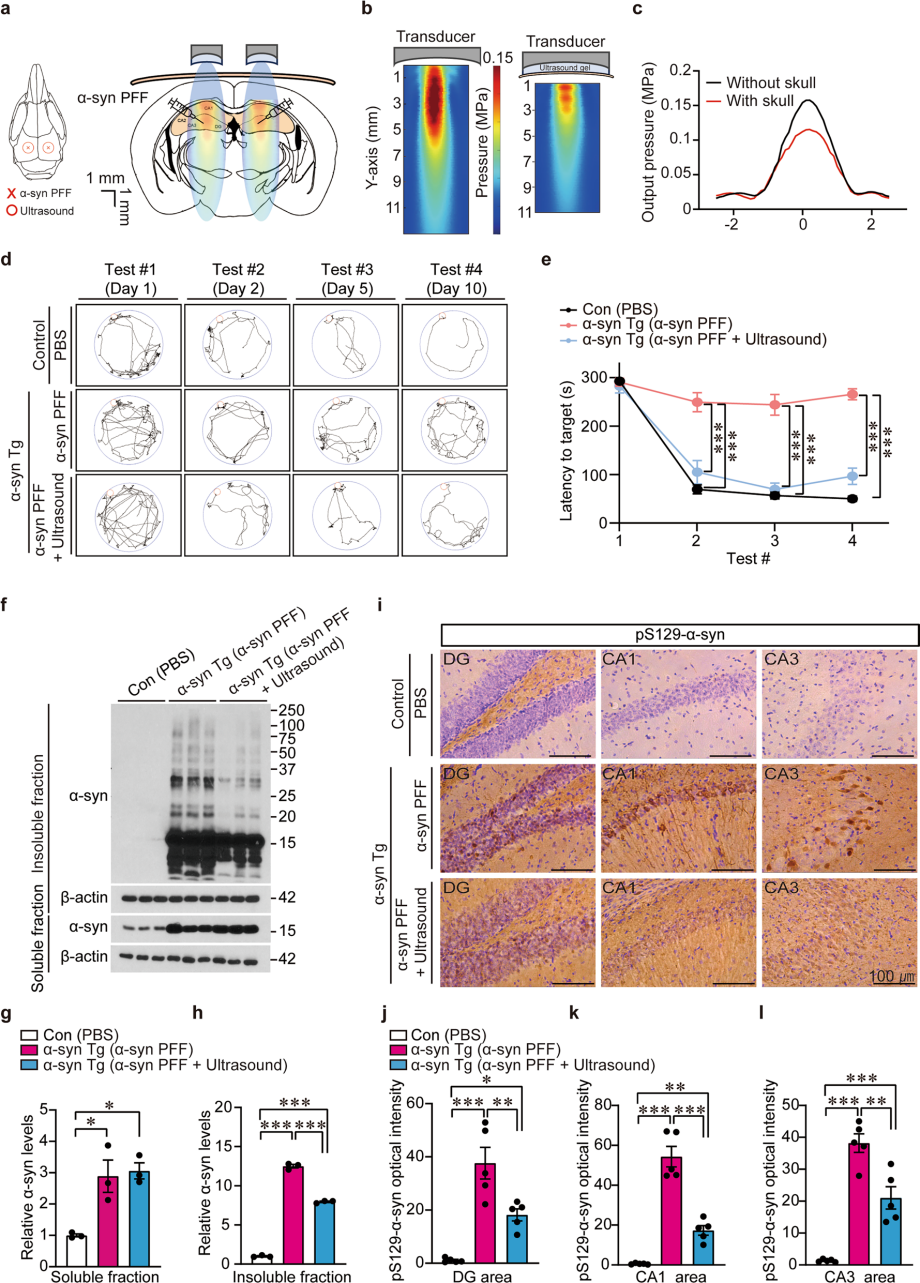
Transcranial ultrasound application prevents cognitive impairments and Lewy pathology in mice with hippocampal α-syn PFF injection. **a** Overall schematic diagram of mouse brain anatomy showing the site of α-syn PFF injection (5 μg/μL, each site 2 μL) and transcranial stimulation region using focused ultrasound. The detailed experimental schedule is shown in Fig. S5a. **b** Ultrasound beam profile with and without mouse skull, measured using needle hydrophone. **c** Comparison of the measured acoustic output pressure level at the focus region with and without mouse skull. **d** Representative exploratory paths of each mouse in the circular arena of the Barnes maze in repetitive trials (days 1, 2, 5, and 10). **e** Assessment of spatial learning and memory of each experimental group as determined by the time taken to find the escape hole in repeated trials in the Barnes maze behavior test (*n* = 10 mice per group). **f** Western blot analysis of α-syn aggregation pathology in Triton X-100-insoluble and -soluble protein fractions of hippocampi from α-syn transgenic mice or age-matched control that received hippocampal injections of α-syn PFF (each site 2 μL [5 μg/μL], 30 days) or PBS as a vehicle, respectively. Ultrasonic application was performed as shown in **a** and **b** to stimulate hippocampal brains. β-Actin served as the internal loading control. **g, h** Quantification of α-syn protein level in the Triton X-100-soluble and -insoluble fractions (*n* = 3 per group). **i** α-Syn pathologies in the dentate gyrus (DG), CA1, and CA3 subregions of hippocampus. **j–l** Quantification of pS129-α-syn expression in the DG, CA1, and CA3 subregions of the indicated mouse groups (*n* = 5 mice per group). All data are presented as mean ± standard error of the mean. *P < 0.05, **P < 0.01, and ***P < 0.001, one-way analysis of variance test followed by Tukey’s post hoc analysis. Full, uncropped original images corresponding to panel **i** are provided in Fig. S8−1

We created the LBD mouse model by stereotaxic injection of α-syn PFFs into the bilateral hippocampal dentate gyrus (DG) regions of α-syn (A53T) transgenic mice (Fig. 4a). As a baseline, we characterized potential background neuropathologies in the α-syn (A53T) transgenic mice. Neuronal expression of A53T α-syn was induced at 1 month of age by the CamKIIα-tTA driver, resulting in robust expression throughout hippocampal neurons (Fig. S3a). However, by 3 months of age, these transgenic mice did not exhibit detectable neuroinflammation (astrogliosis or microgliosis), pS129-αSyn-positive inclusions, or hippocampal neuronal loss in the DG and CA1 regions (Fig. S3b–f). Consistent with the absence of neuropathology, the α-syn Tg mice displayed normal motor and cognitive functions compared with littermate controls (Fig. S3g–j). These results indicate that the α-syn Tg mice provide a suitable background for hippocampal PFF inoculation to model α-syn seeding and propagation under prodromal conditions of high neuronal α-syn expression. To accelerate α-syn pathology formation and propagation, α-syn PFFs were injected into the brains of the α-syn Tg mice [38]. The control group consisted of CamKIIα-tTA driver mice stereotaxically injected with PBS.

Before applying ULIUS (0.2 W/cm^2^) in vivo, we first assessed its potential adverse effects in wild-type mouse brains. For comparison, we also tested a stronger ultrasound paradigm (2.5 W/cm^2^), previously reported to exert anti-inflammatory effects in an ischemic stroke model with short-term application [39]. Repeated transcranial ULIUS targeted to the hippocampus for one month produced no detectable pathological changes compared with sham controls (Fig. S4a–g). In contrast, long-term application of 2.5 W/cm^2^ focused ultrasound (10 sessions over one month) led to marked reductions in DG thickness and significant hippocampal neuron loss in the DG (Fig. S4a–c). This was accompanied by robust increases in the percentage area fractions of Iba-1 and GFAP signals in both DG and CA1 subregions (Fig. S4d–f), indicating neuroinflammation consistent with underlying neurodegeneration. Prominent infiltration of Iba-1-positive microglia into the DG was also observed following 2.5 W/cm^2^ ultrasound treatment (Fig. S4g), further supporting ongoing neuroinflammation in this region. Since long-term ULIUS did not cause adverse neuropathological effects in wild-type mice, we next applied this protocol to α-syn transgenic mice with hippocampal PFF injections to evaluate its therapeutic potential.

Ultrasound was delivered over the injected regions of the hippocampus across the intact mouse skull using a custom-made 1 MHz focused single-element transducer with a focal distance of 3 mm. It was repeated 10 times every 3 days (Fig. S5a). As confirmed by hydrophone measurements, the focused beam shape was maintained with the intact skull (Fig. 4b). The post-cranial pressure became 0.11 MPa, which could induce astrocyte-specific stimulations (Fig. 4c) as shown in the previous study [38]. Owing to the limited penetration of 6 MHz, the frequency was lowered to 1 MHz. Other transmission parameters such as pulse length, pulse repetition rate, and duty cycle were the same as those for the in vitro experiments.

Barnes maze behavior test was performed to assess memory and cognitive abilities in control and α-syn Tg mice after a month of the indicated treatments following stereotaxic surgery (Fig. S5a). In contrast to intact spatial learning and memory in control mice, α-syn Tg mice injected with α-syn PFFs displayed a ~ 300-s delay to reach the target hole in all repeat trials (Fig. 4d, e), indicating loss of cognitive learning and memory functions. Transcranial-focused ultrasound application substantially improved cognitive functions of the LBD mouse model to be comparable with the littermate controls, as evidenced by the repeat trials of Barnes maze (Fig. 4d, e). Our LBD mouse model also exhibited short-term working memory deficits, evidenced by reduced alternation triplets in the elevated Y-maze, which were rescued by transcranial ULIUS treatment (Fig. S5b).

α-Syn PFF injection into mouse brains induces endogenous α-syn aggregate formation via seed-template mechanisms and propagation from the injection site to other connected brain regions [5, 40]. Consistent with these previous studies, hippocampal injection of α-syn PFFs into α-syn Tg mice led to robust formation of detergent-insoluble α-syn aggregates in the hippocampus (Fig. 4f–h). This enhancement of α-syn aggregation was markedly reduced by transcranial ultrasound application, although the total soluble α-syn expression in transgenic mice was not altered by ultrasound (Fig. 4f, g). Consistent with western blot analysis, transcranial ultrasound treatment significantly prevented the formation of pS129-α-syn-positive Lewy-like inclusions in the LBD mouse model in all the subregions of hippocampus (Fig. 4i–l). We also observed α-syn pathology propagation to the cortex at 1 month after hippocampal injection of α-syn PFFs in transgenic mice (Fig. S5c, d). The pS129-α-syn signals in the cortex of LBD mouse model were considerably suppressed by ultrasound treatment (Fig. S5c, d), indicating that both the formation and the propagation of α-syn aggregates were effectively blocked by transcranial ultrasound application. In contrast to the marked propagation of Lewy-like pathology to the cortex in our LBD model, no spread of α-syn pathology to the ventral midbrain was observed in three-month-old α-syn Tg mice that received hippocampal αSyn PFF injections at two months of age, despite robust seeding in the hippocampal regions (Fig. S5e, f). Consistent with the absence of ventral midbrain pathology, we did not detect dopaminergic neuron loss or astrogliosis (Fig. S5g–j). Moreover, α-syn Tg mice with hippocampal PFF injections retained normal motor function, comparable to control mice, as assessed by the pole test and accelerating rotarod (Fig. S5k, l).

### Ultrasound-mediated brain stimulation blocks the α-syn PFF-induced TRPA1/TLR2 expression and prevents neuroinflammation and neurodegeneration in α-syn transgenic mice with hippocampal injection of α-syn PFFs

**Fig. 5:**
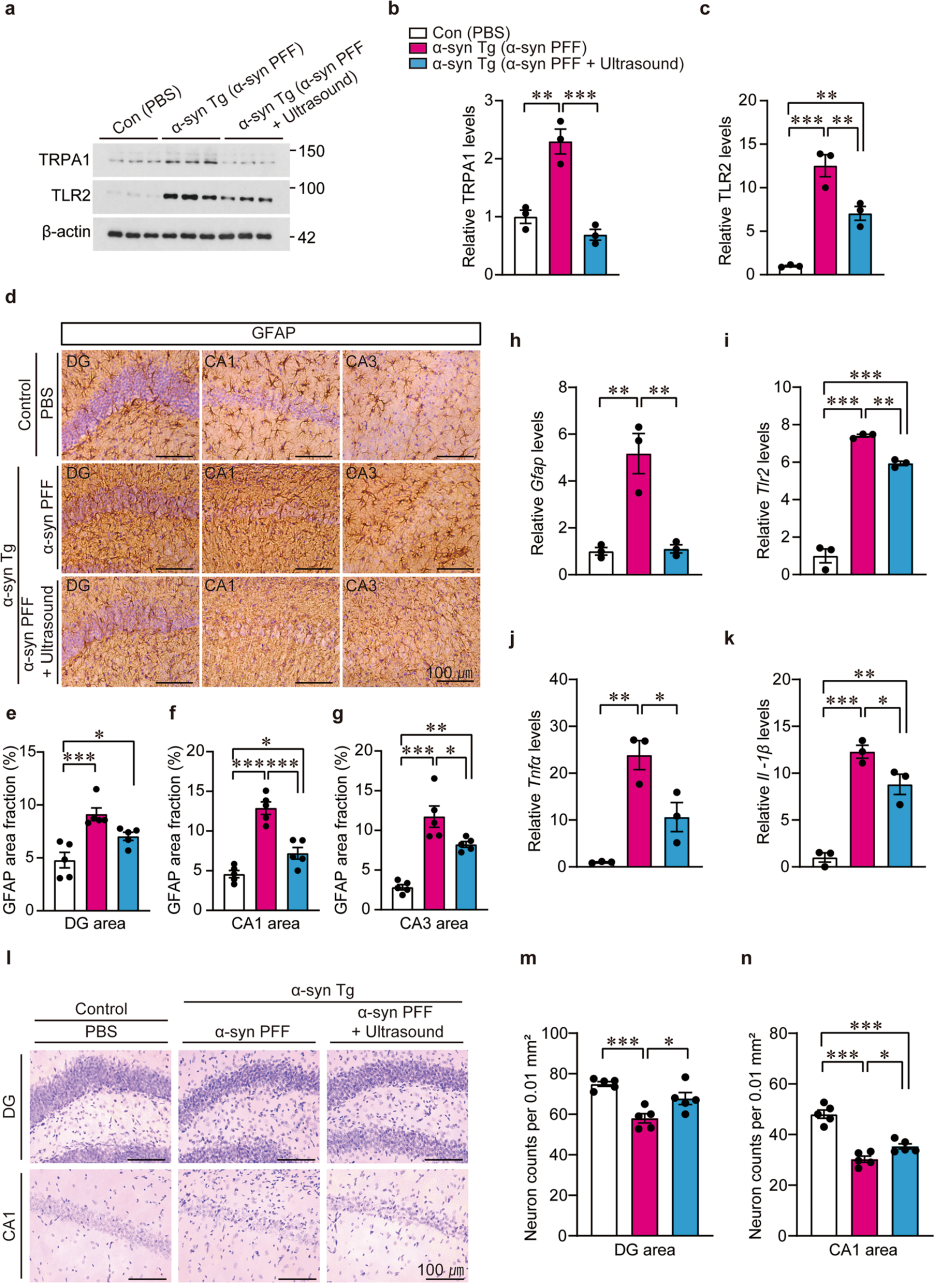
Transcranial ultrasound application in mice with hippocampal α-syn preformed fibril (PFF) injection suppresses TLR2 and TRPA1 expression, mitigates inflammatory cytokine production, and prevents hippocampal neurotoxicity. **a** Western blot analysis of TRPA1 and TLR2 protein levels in hippocampal brain lysates from control and α-syn transgenic mice that received brain injections of PBS or α-syn PFFs (each site 2 μL [5 μg/μL], 30 days). Ultrasonic application was performed as shown in panels **a** and **b** of Fig. 4. β-Actin served as the internal loading control. **b, c** Quantification of protein levels of TRPA1 and TLR2 (*n* = 3 mice per group). **d** Astrogliosis in the dentate gyrus (DG), CA1, and CA3 subregions of hippocampus in the indicated mouse groups by immunohistochemisty. The brain sections were counterstained with Nissl. **e–g** Quantification of GFAP signal intensities in the DG, CA1, and CA3 subregions (*n* = 5 mice per group). **h**–**k** Quantification of mRNA expression of *Gfap, Tlr2, Tnfα*, and *Il-1β* in the cortex/hippocampus, as assessed by RT-PCR (*n* = 3 per group). **l** Representative images of Nissl-stained coronal sections of DG and CA1 from the indicated experimental mouse groups. **m, n** Nissl-stained neuronal cell counts in the DG or CA1 subregions of the hippocampus from each experimental mouse group (*n* = 5 mice per group). All data are presented as mean ± standard error of the mean. *P < 0.05, **P < 0.01 and ***P < 0.001, one-way analysis of variance test followed by Tukey’s post hoc analysis. Full, uncropped original images corresponding to panels **d** and** l** are provided in Fig. S8−1

Based on our in vitro ultrasound and astrocyte modulation findings, we investigated neuroinflammation-associated cellular and molecular profiles as the underlying mechanisms for the ultrasound-mediated therapeutic effects on α-synucleinopathy in mice. Consistent with the astrocytic expression of *Trpa1* and *Tlr2* mRNA in response to α-syn PFF treatment (Fig. 3d, h, i), there was an over two-fold increase in TRPA1 and an approximately 12-fold increase in TLR2 protein level in the α-syn PFF-injected hippocampi of transgenic mice (Fig. 5a–c). Transcranial ultrasound stimulation of the hippocampus completely reversed TRPA1 expression to the basal level and induced an approximately 50% reduction of TLR2 expression in the α-syn PFF-injected transgenic mice (Fig. 5a–c). The ultrasound-induced reduction of TLR2 was validated in hippocampal subregions by immunofluorescence (Fig. S6a–c).

To determine whether the modulation of TLR2 and TRPA1 expression is associated with astrocyte reactivity, anti-glial fibrillary acidic protein (GFAP) immunohistochemistry was performed. Correlating with α-syn aggregate pathology distribution, enhanced GFAP signals were observed in the hippocampal subregions and the cortex of α-syn transgenic mice with hippocampal α-syn PFF injections (Fig. 5d–g and Fig. S6d, e). Transcranial ultrasound treatment mitigated neuroinflammation in all hippocampal subregions and alleviated the spread of inflammation to the cortex (Fig. 5d–g and Fig. S6d, e). Consistent with anti-GFAP immunohistochemistry, the upregulation of *Gfap* transcripts in the dissected brains (hippocampus and cortex) of the LBD mouse model was almost entirely prevented by ultrasound (Fig. 5h). The elevated levels of *Tlr2, Tnfα,* and *Il-1β* mRNAs in the hippocampus and cortex of α-syn PFF-injected transgenic mice were significantly decreased by transcranial ultrasound treatments (Fig. 5i–k), indicating the modulation of inflammatory processes by ultrasound in vivo. In addition to astrogliosis and inflammation in LBD mouse brains, there was a marked increase in Iba1 immunohistochemical signals (Fig. S6f–j), indicating microglial activation. This microgliosis was effectively suppressed by transcranial ultrasound (Fig. S6f–j). Neuroinflammation (astrogliosis, and microgliosis) was accompanied by neurodegeneration in the hippocampus, DG, CA1, and cortex, as determined by stereological Nissl-stained neuron counts in the corresponding subregions (Fig. 5l–n, and Fig. S6k, l). Transcranial ultrasound provided varied levels of neuroprotection in all the tested brain subregions, with maximum protection in DG-resident neurons (Fig. 5l–n, and Fig. S6k, l). Taken together, these results suggest that transcranial ultrasound application modifies the α-syn-induced astrocytic and microglial pathologic transitions, potentially through regulation of TRPA1 and TLR2 expression and function.

## Discussion

### ULIUS brain stimulation as a disease-modifying therapy for brain diseases with formation and propagation of α-syn pathology

In this translational study, we demonstrated that transcranial focused ULIUS targeting the hippocampus has disease-modifying effects, offering neuroprotection and suppressing inflammation in the brains of LBD mouse model (Fig. S7), preventing the development of cognitive impairment. Although some studies have presented the disease-modifying effects of ultrasound in PD mouse models and α-syn transgenic mice, its translational therapeutic efficacy has not been evaluated in α-syn PFF-induced LBD mouse model, in which α-syn and inflammation propagate through the hippocampus and cortex. As α-syn pathology can propagate throughout connected brain regions and induce the spread of neurotoxicity and inflammation, ultrasound holds great promise for preventing the initial formation of α-syn aggregates and suppressing the pathological transmission of α-synucleinopathy to other brain regions. Indeed, patients with PD dementia experience motor symptoms as α-syn pathologies affect ventral midbrain dopaminergic neurons several years prior to cognitive impairment. Ultrasound has the advantages of high spatial resolution and deep brain penetration, compared with other noninvasive or pharmacologic neuromodulatory technologies. We and other groups have shown effective stimulation of the hippocampus and deep brain structures, such as the substantia nigra in mice [41–43]. The protective effects of ULIUS in the α-syn PFF model are best explained by its ability to suppress multicellular pathogenic processes initiated by extracellular α-syn fibrils and oligomers. Notably, previous studies demonstrated that targeting glial activation alone was sufficient to block a broad spectrum of α-syn-induced pathologies, including neuronal degeneration, pathological spreading, inflammation, and cognitive impairment. For instance, GLP-1 receptor activation by NLY-01 prevented microglia-mediated conversion of astrocytes to an A1 neurotoxic phenotype and conferred neuroprotection in α-syn PFF models [34]. Likewise, inhibition of TLR2 signaling or treatment with anti-inflammatory drugs (indomethacin, ibuprofen) protected against α-syn oligomer–induced memory impairment in mice [6]. Together with our findings, these studies underscore that glial inhibition—particularly suppression of astrocyte-driven inflammation—is sufficient to halt the cascade of multicellular pathologies in α-synucleinopathy, highlighting the critical role of astrocytes in disease progression. This suggests that when α-syn pathology is located in peripheral organs or in certain brain subregions, focused ultrasound could be implemented to suppress not only the existing neuroinflammation at the target site but also the ongoing propagation of α-syn pathologies. Moreover, given that astrogliosis and neuroinflammation are involved in many neurodegenerative disorders, transcranial ultrasound may have broader therapeutic applications for diverse brain diseases. Future studies employing additional α-syn transgenic mouse models that recapitulate Lewy body-associated neuropathologies and behavioral deficits will be essential to validate and generalize the therapeutic potential of ultrasound stimulation, extending its translational impact beyond a single novel LBD model.

Although long-term human data are still limited, studies of low-intensity transcranial focused ultrasound neuromodulation in healthy volunteers have reported no serious adverse events, with only transient and reversible symptoms [44–46], and consensus guidelines have defined safe biophysical limits for clinical use. Consistent with this, we observed no pathological alterations after repeated 0.2 W/cm^2^ ULIUS in wild-type mice for one month, whereas higher-intensity (2.5 W/cm^2^) ultrasound caused hippocampal neurodegeneration and inflammation, underscoring the importance of intensity in long-term applications. Importantly, long-term hippocampal-focused ULIUS not only reduced neuroinflammation but also suppressed the propagation of α-syn aggregates from the hippocampus to cortical regions in our LBD model, suggesting a preventive effect by limiting the spread of pathology across connected brain networks. These findings support ULIUS as a safe and promising paradigm with both therapeutic and preventive benefits for LBD. At the same time, because astrocytic TRPA1 contributes to redox sensing, neurovascular coupling, and synaptic plasticity [47–50], chronic inhibition may carry risks, highlighting the need for further preclinical and clinical studies to establish the long-term safety and translational potential of ULIUS.

### Molecular mechanisms of ultrasound modulation of astrocyte reactivity in response to α-syn PFF treatment

Astrocytic TRPA1 is a molecular target of ULIUS. It has been shown that the ULIUS-mediated selective activation of TRPA1 in astrocytes under basal condition is responsible for glutamate release and subsequent firing of neighboring neurons via the *N*-methyl-*D*-aspartate receptor [14]. The brief ultrasound-induced increase in intracellular calcium levels in astrocytes is mediated by TRPA1 under physiological conditions [14]. Our study demonstrated for the first time that ultrasound application to α-syn PFF-treated inflammatory astrocytes suppresses intracellular calcium levels over time (Fig. 3), despite an initial transient increase in calcium, similar to the observations from control astrocytes under physiological conditions. The reason for the different intracellular calcium responses of astrocytes under physiological conditions and those under α-syn PFF–treated pathological conditions to the same ultrasound stimulation remains unclear. The inflammatory signaling initiated by α-syn-stimulated TLR2 and TRPA1-mediated sustained intracellular calcium may influence the post-translational modifications of TRPA1, thus modulating its response to mechanical stimuli from ultrasound. Further studies are required to elucidate the underlying molecular mechanisms of ultrasound-mediated differential modulation of TRPA1 activity under physiological versus pathological conditions in PD. We demonstrated that ultrasound prevented the pathogenic conversion of astrocytes in response to α-syn PFF treatment. In this study, inflammation and neurotoxicity-relevant molecular profiles in astrocytes were examined mainly through RT-qPCR assessment of a certain set of transcripts. As ultrasound exerts differential modulation of calcium signals in the α-syn PFF astrocytes, unbiased whole transcriptomic profiling would enable a better understanding of the effect of ultrasound on the phenotypic transformation of astrocytes in PD-associated pathological environments.

### Roles of TRPA1-TLR2 in neuroinflammation and α-synucleinopathy of LBD and their potential as therapeutic targets

In this study, we found that chronic exposure of astrocytes to α-syn PFFs induced transcriptional activation of both *Trpa1* and *Tlr2*, which corresponded to sustained calcium influx in response to acute α-syn PFF treatment. Elevated TRPA1 expression, upon chronic exposure to α-syn PFF, mediated increased calcium influx and astrocyte hyperactivation. Conversely, as a receptor for α-syn PFF, enhanced TLR2 expression contributed to the sensitized response of astrocytes to α-syn PFF treatment, indicating a feed-forward acceleration of astrocyte pathogenic conversion. Characteristic calcium signaling in response to α-syn PFFs in astrocytes appears to be required for this transcriptional regulation, as pharmacological or ultrasound disturbance of calcium signaling blocked the α-syn-induced elevation of TRPA1 and TLR2. Nuclear factor kappa B (NF-kB) activation may drive astrocytic production of proinflammatory cytokines under α-syn PFF exposure, as NF-kB is downstream of TRPA1 and TLR2 signaling pathways [26, 51]. Expression of both TRPA1 and TLR2 has been shown to be regulated by the proinflammatory cytokine TNFα under pathological conditions [52, 53]. Neuronal and glial expression of TLR2 and proinflammatory cytokines are increased in postmortem brains of patients with PD [35, 54], supporting the clinical relevance of TLR2 in α-synucleinopathy. Our study was the first to report increased TRPA1 and TLR2 expression in the hippocampus of LBD mice caused by α-syn PFF injection. Postmortem studies of regional and cellular distribution and expression of TRPA1 in the brains of patients with α-synopathies are needed to determine the clinical relevance of TRPA1 in LBD.

Pharmacological inhibition of TRPA1 prevents amyloid plaque accumulation, astrocyte hyperactivation, and neuronal dysfunction, thereby ameliorating cognitive impairment in AD model mice [31]. Immunological intervention of TLR2 signaling provides neuroprotection in α-syn transgenic mice [35]. Notably, ULIUS every 3 days for a month was sufficient to repress the α-syn PFF-induced TRPA1 and TLR2 expression. Intermittent ultrasound application eventually induced long-lasting alterations and disease modifications both in vitro and in vivo. Compared with small compound inhibitors of TRPA1 or TLR2 antibodies that lack regional selectivity and have poor brain distribution, the non-invasive and safe ultrasound application has the advantage of focused therapeutic modulation of α-syn pathology and neuroinflammation in targeted brain regions. Alternative ultrasound parameters could be evaluated for improved safety and therapeutic efficacy in our accelerated LBD dementia mouse model and other established models.

## Conclusions

We demonstrated that repeated low-intensity transcranial ULIUS is safe for long-term application, whereas stronger, prolonged ultrasound may cause hippocampal inflammation and neurodegeneration. Building on this safety profile, our study further established the translational therapeutic potential of focused transcranial ULIUS in preventing the initiation and spread of α-syn aggregate pathology and neuroinflammation in a Lewy body dementia mouse model. Our findings highlight the critical role of sustained TRPA1 activation and calcium influx in driving the reactivation of astrocytes in response to α-syn PFF treatment. Specifically, TRPA1 on astrocytes acts upstream of inflammatory signaling pathways, regulating the expression of the α-syn PFF receptor TLR2 and pro-inflammatory cytokines. Notably, focused ultrasound mitigates these pathogenic signaling pathways by repressing TRPA1 activity under α-syn-induced pathological conditions.

## Supplementary Information

Additional file 1. **Fig. S1**. Information on Ultrasonocoverslip. **Fig. S2**. Ultrasound and TRPA1 inhibition suppress astrocytic inflammatory responses induced by conditioned media from α-synuclein PFF–exposed BV2 cells and by direct PFF treatment, as measured by RT-qPCR. **Fig. S3**. Conditional α-synuclein (A53T) Tg transgenic mice have no neuropathologies in brains with normal behavior. **Fig. S4**. Ultrasound intensity-dependent brain pathologies in wild type mice. **Fig. S5**. Hippocampal PFF injection in α-synuclein transgenic mice induces Lewy pathology spread to the cortex, but not to the ventral midbrain, within 1 month. **Fig. S6**. Transcranial ultrasound application represses hippocampal TLR2 upregulation, microgliosis, and neurodegeneration in α-synuclein transgenic mice with hippocampal α-syn preformed fibril (PFF) injection.** Fig. S7**. Schematic illustration of ultrasound-mediated astrocyte repression and neuroprotection in LBD brains. **Fig. S8-1**. Full, uncropped original brain section images corresponding to main and supplementary figure panels. **Fig. S8-2.** Full, uncropped original brain section images corresponding to main and supplementary figure panels. **Table S1**. Sequences of primers used for RT-qPCR.

Additional file 2. Full blots.

## Data Availability

All data required to evaluate the conclusions of this study are presented in the paper and/or Supplementary Materials. Any additional data request can be made to the corresponding authors.

## Abbreviations

LBD: Lewy body dementia
ULIUS: Ultra-low-intensity ultrasound
TRPA1: Transient receptor potential ankyrin 1
TLR2: Toll-like receptors-2
PFF: Preformed fibril
LIFUS: Low-intensity focused ultrasound stimulation
TNF-α: Tumor necrosis factor-alpha
IL-1β: Interleukin-1 beta
RT-PCR: Reverse-transcription polymerase chain reaction
MAP2: Microtubule-associated protein 2
MCM: Microglia conditioned media
ACM: Astrocyte conditioned media
DG: Dentate gyrus
