# Supplementary material for: Ultrasonic repression of TRPA1-dependent astrocyte reactivity confers neuroprotection in models of Lewy body dementia
Source: Transl Neurodegener. 2026 Mar 10;15:9. doi: 10.1186/s40035-026-00544-6 (PMC12973804; doi:10.1186/s40035-026-00544-6)
Supplement: Supplementary file 1 — Additional file 1. Fig. S1. Information on Ultrasonocoverslip. Fig. S2. Ultrasound and TRPA1 inhibition suppress astrocytic inflammatory responses induced by conditioned media from α-synuclein PFF–exposed BV2 cells and by direct PFF treatment, as measured by RT-qPCR. Fig. S3. Conditional α-synuclein (A53T) Tg transgenic mice have no neuropathologies in brains with normal behavior. Fig. S4. Ultrasound intensity-dependent brain pathologies in wild type mice. Fig. S5. Hippocampal PFF injection in α-synuclein transgenic mice induces Lewy pathology spread to the cortex, but not to the ventral midbrain, within 1 month. Fig. S6. Transcranial ultrasound application represses hippocampal TLR2 upregulation, microgliosis, and neurodegeneration in α-synuclein transgenic mice with hippocampal α-syn preformed fibril (PFF) injection. Fig. S7. Schematic illustration of ultrasound-mediated astrocyte repression and neuroprotection in LBD brains. Fig. S8-1. Full, uncropped original brain section images corresponding to main and supplementary figure panels. Fig. S8-2. Full, uncropped original brain section images corresponding to main and supplementary figure panels. Table S1. Sequences of primers used for RT-qPCR. [file 40035_2026_544_MOESM1_ESM.pdf]

Translational Neurodegeneration

Ultrasonic repression of TRPA1-dependent astrocyte reactivity confers neuroprotection in models of Lewy body dementia

Ji Hun Kim[1,†] , Keunhyung Lee[2,†], Minseok Koo[2] ,Doeun Kim[1], Jin Kyung Hong[1], Jeong-Yun Choi[1], Han Seok Ko[3], Joo-Ho Shin[1], Joo Min Park[4,5], Jinhyoung Park[2,6,*], Yunjong Lee[1,*]

[1]Department of Pharmacology, Sungkyunkwan University School of Medicine, Samsung Biomedical Research Institute (SBRI), Suwon 16419, Republic of Korea.

[2]Department of Intelligent Precision Healthcare Convergence, Sungkyunkwan University, Suwon, Republic of Korea

[3]Neuroregeneration and Stem Cell Programs, Institute for Cell Engineering, Johns Hopkins University School of Medicine, Baltimore, MD 21205, USA.

[4]Center for cognition and Sociality Institute for Basic Science (IBS) Daejeon Korea

[5]University of Science and Technology (UST), Daejeon, Republic of Korea.

[6]Department of Biomedical Engineering, Sungkyunkwan University, Suwon, Republic of Korea

[†] These authors contributed equally to this work

[*] Corresponding author

Correspondence should be addressed to:

Jinhyoung Park, PhD

Department of Intelligent Precision Healthcare Convergence

Department of Biomedical Engineering

Sungkyunkwan University

300 Cheoncheon-dong, Jangan-gu

Suwon, Gyeonggi-do 440-746, Republic of Korea

Email: jin.park@skku.edu

Yunjong Lee, PhD

Department of Pharmacology

Sungkyunkwan University School of Medicine

300 Cheoncheon-dong, Jangan-gu

Suwon, Gyeonggi-do 440-746, Republic of Korea

Email: ylee69@skku.edu

SUPPLEMENTARY FIGURES and LEGENDS

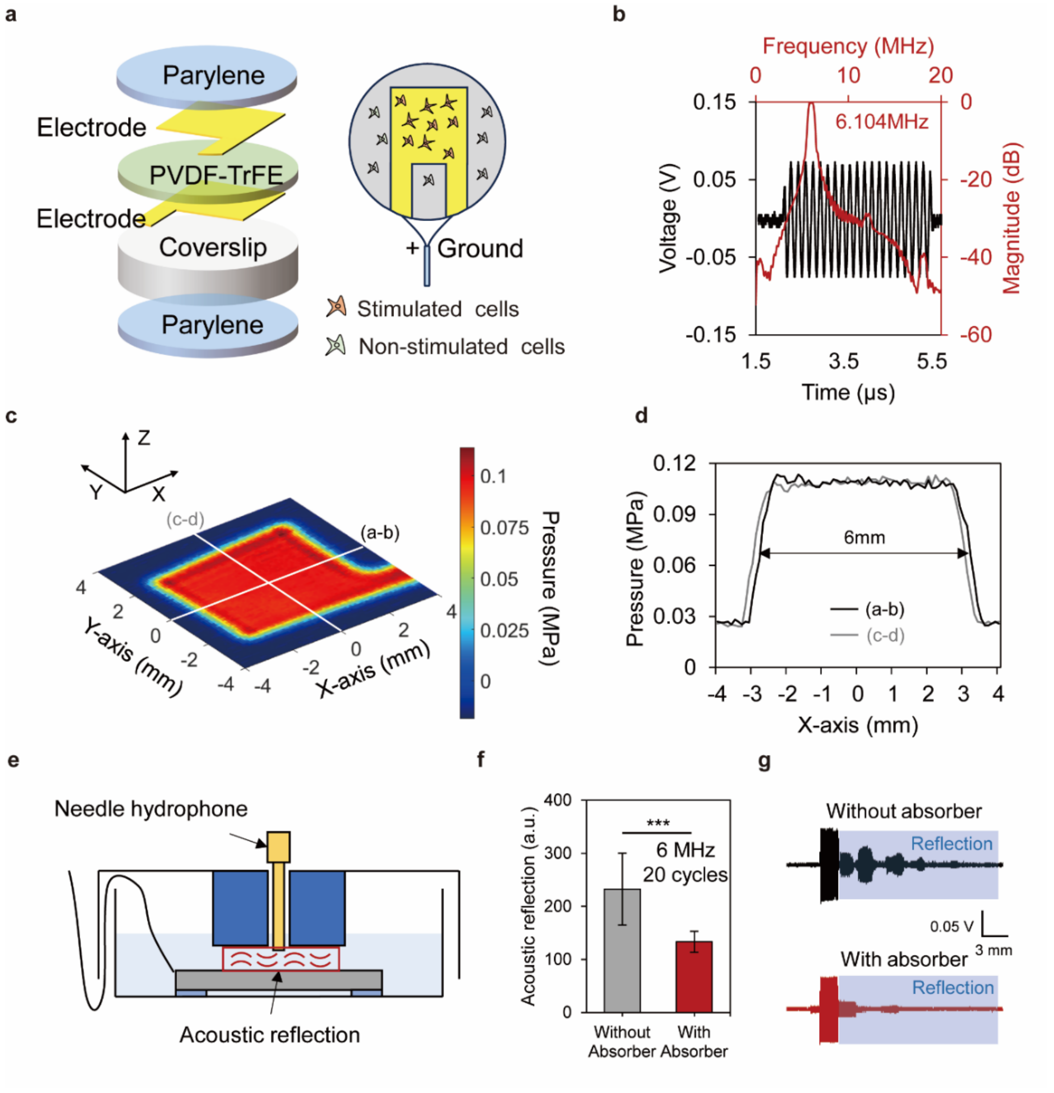

Fig. S1. Information on Ultrasonocoverslip. a Structure of Ultrasonocoverslip. The dimension of ultrasound active area is 6 mm × 6 mm with a square shape. The thickness of the glass-coverslip, PVDF-TrFE, and parylene layer is 170 µm, 16 µm, and 12 µm respectively. **b** Pulse-echo result of Ultrasonocoverslip. Fundamental frequency was measured as 6.104 MHz with 20 cycles of ultrasound pulses. **c** Acoustic beam profile measured in the XY direction, perpendicular to the surface of the Ultrasonocoverslip.

Measured averaged acoustic output pressure in ultrasound active area was 0.11 MPa. **d** Line plots along with (**a, b**) and (**c, d**) line noted in Figure. S1**c**. **e** Configuration of acoustic reflection measurement. **f, g** Calculated acoustic reflection triggered by 6 MHz and 20 cycles of ultrasound pulses through a needle hydrophone with and without absorber.

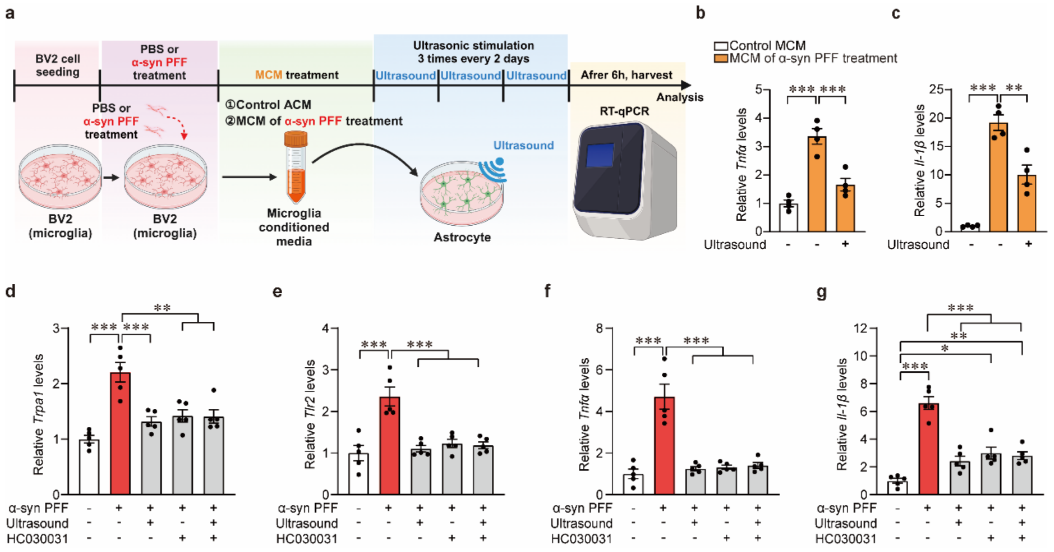

Fig. S2. Ultrasound and TRPA1 inhibition suppress astrocytic inflammatory responses induced by conditioned media from α-synuclein PFF–exposed BV2 cells and by direct PFF treatment, as measured by RT-qPCR. a Scheme of experimental procedures for repeated ultrasound applications to primary astrocyte cultured with conditioned media from BV2 cells treated with α-synuclein PFF or phosphate-buffered saline as a vehicle. At the completion of scheduled treatments, the astrocytes were subjected to real-time quantitative polymerase chain reaction (qPCR) to examine the expression of inflammatory cytokines. **b,c** Quantification of messenger RNA levels for *Tnfα* and *Il-1β* in primary astrocytes cultured in the conditioned media (7 days) prepared from BV2 cells treated with/without α-synuclein PFF (5 µg/mL, 1 day) as determined using real-time qPCR ($n = 4$ per group). Ultrasound was repeatedly applied to astrocytes as indicated in Panel A. *Gapdh* was used as the internal loading control for normalization. **d-g** Real-time qPCR analysis of *Trpa1, Tlr2, Tnfα*, and *Il-1β* mRNA levels in primary astrocytes treated with α-synuclein PFFs (5 µg/mL, 5 h) to model α-synucleinopathy, with or without TRPA1 inhibition (HC030031, 10 µM) and/or

ultrasound treatment applied every two days for a total of three treatments (n = 5 separate experiments per group). *Gapdh* was used as the internal loading control for normalization. Data in all panels are presented as mean ± standard error of the mean. *$P < 0.05$, **$P < 0.01$ and ***$P < 0.001$, one-way analysis of variance test followed by Tukey's *post hoc* analysis.

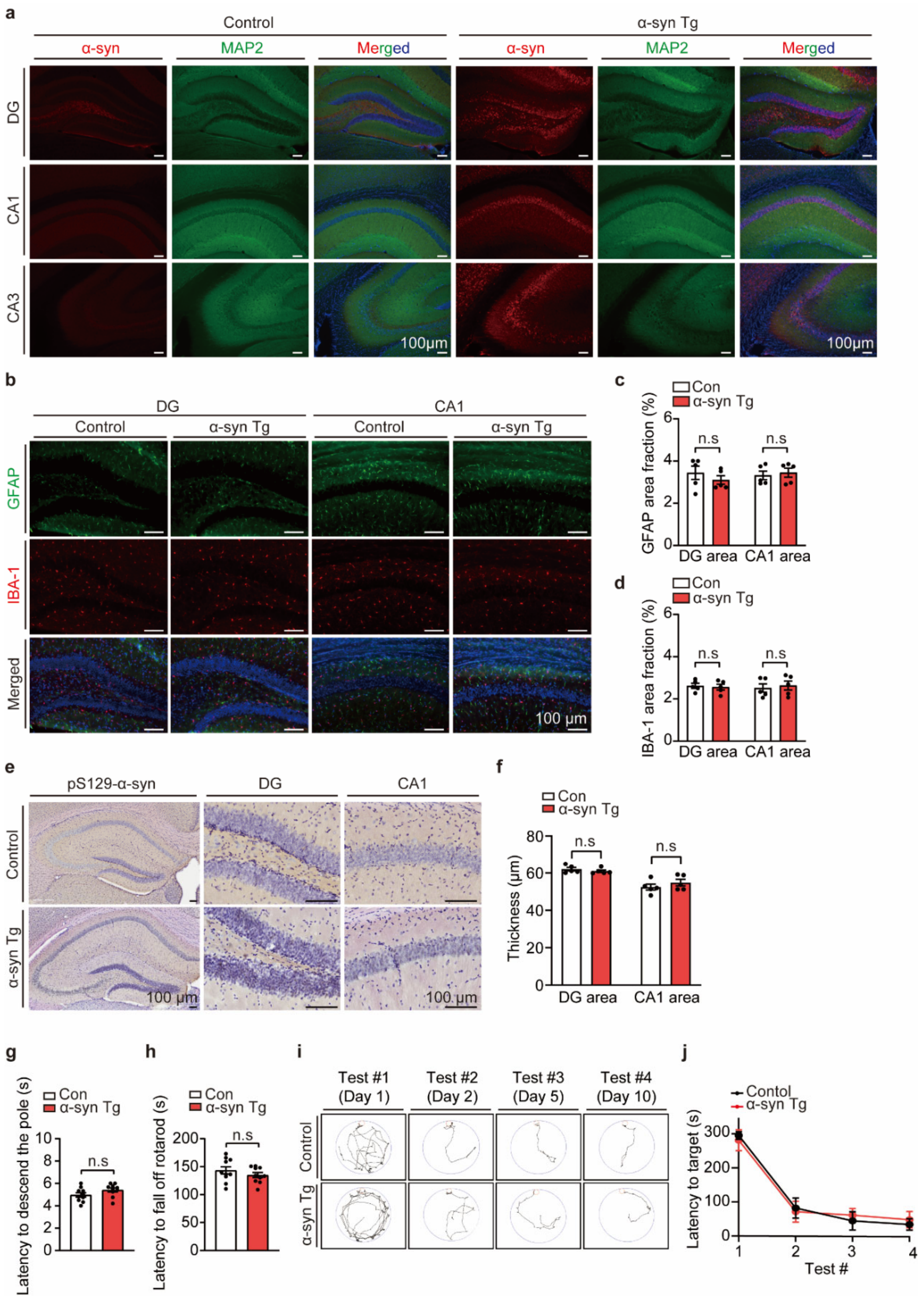

Fig. S3. Conditional α-synuclein (A53T) Tg transgenic mice have no neuropathologies in brains with normal behavior. a Immunofluorescence staining of hippocampal sections from conditional α-synuclein transgenic mice and littermate

control showing expression pattern of α-synuclein. MAP2 was used for neuron labeling. α-synuclein transgene expression was induced from 1 month to 3 month of age. Scale bar = 100 μm. **b** Representative anti-GFAP and anti-IBA1 immunofluorescent images of DG and CA1 regions from α-synuclein Tg and littermate control at three months of age. Scale bar = 100 μm. **c, d** Quantification of % area fraction of GFAP and IBA-1 signals in the indicated hippocampal subregions from each mouse group (*n* = 5 mice per group). **e** Representative anti-pS129-αSyn immunohistochemistry of hippocampal brain sections from three month old control and α-synuclein Tg mice. Brain sections were counterstained by Nissl. Scale bar = 100 μm. **f** Quantification of relative thickness of Nissl stained DG, CA1 from each group (*n* = 5 mice per group) . **g, h** Motor functions assessed by Pole and rotarod tests (*n* = 10 mice per group). **i, j** Spatial learning and memory function determined by Barnes maze. Latency to target and representative exploratory paths (*n* = 10 mice per group). Data in all panels are presented as mean ± standard error of the mean. Unpaired two-tailed Student's t-tests. n.s., non-significant. Full, uncropped original images corresponding to figure panels (S3**b**, and S3**e**) are provided in Fig. S8-2.

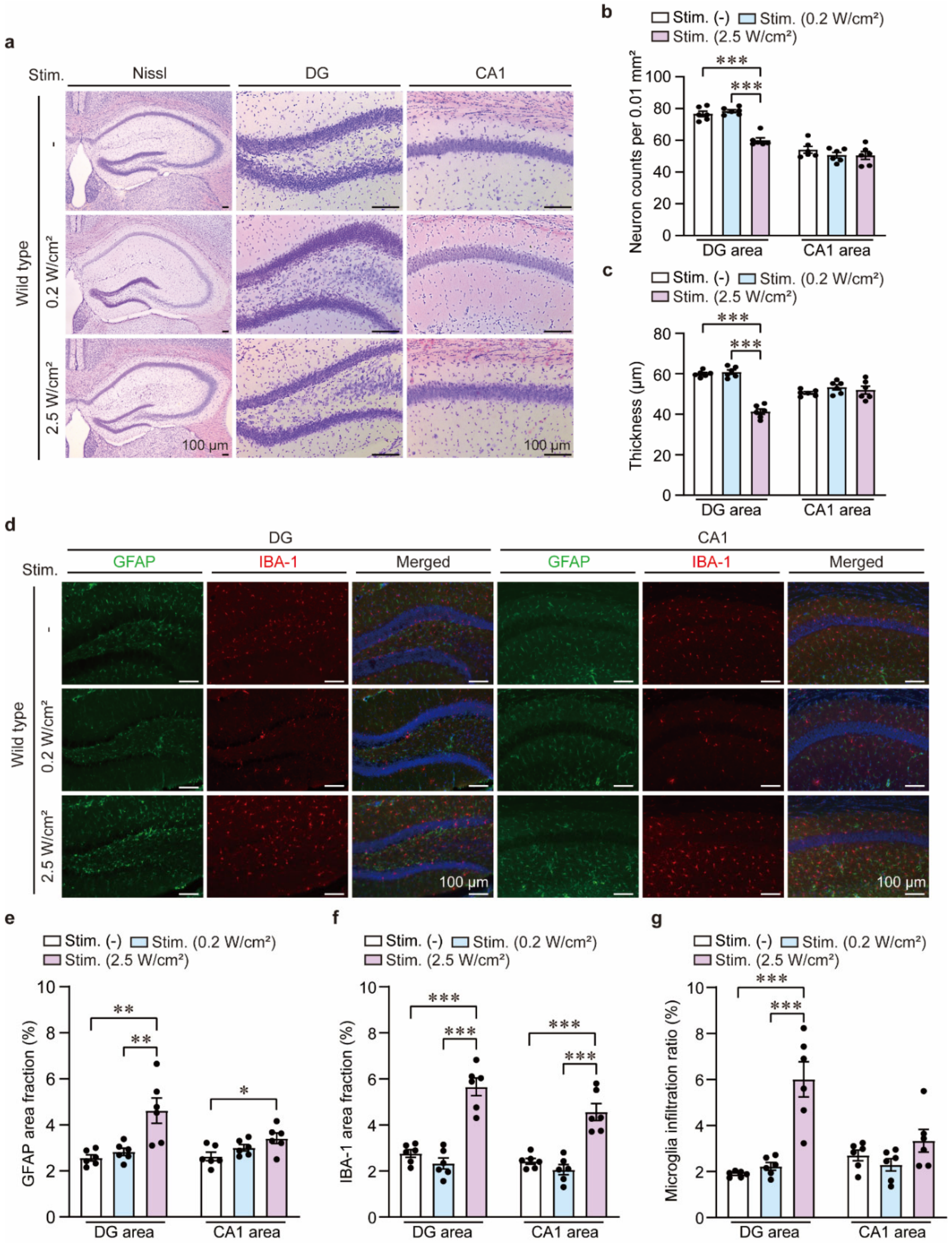

Fig. S4. Ultrasound intensity-dependent brain pathologies in wild type mice. a Representative Nissl-stained hippocampal brain sections from wild-type mice subjected to repeated ultrasound applications (0.2 W/cm² and 2.5 W/cm², 10 sessions total) or no-stimulation controls. Enlarged views of the DG and CA1 subregions are shown. Scale

bar = 100 μm. **b** Quantification of Nissl-stained neuron counts in the DG and CA1 subregions of the indicated groups (*n* = 6 treated hemispheres from 3 mice per group). **c** Quantification of DG and CA1 thickness in the indicated groups (*n* = 6 treated hemispheres from 3 mice per group). **d** Representative images of neuroinflammation in DG and CA1 subregions, assessed by GFAP and IBA-1 immunostaining in the indicated groups. Scale bar = 100 μm. **e,f** Quantification of the % area fraction of GFAP and IBA-1 signals in DG and CA1 subregions (*n* = 6 treated hemispheres from 3 mice per group). **g** Quantification of microglial infiltration ratio in DG and CA1, as determined by IBA-1 signal distribution in the indicated experimental groups (*n* = 6 treated hemispheres from 3 mice per group). Data in all panels are presented as mean ± standard error of the mean. *$P < 0.05$, **$P < 0.01$ and ***$P < 0.001$, one-way analysis of variance test followed by Tukey's post hoc analysis.

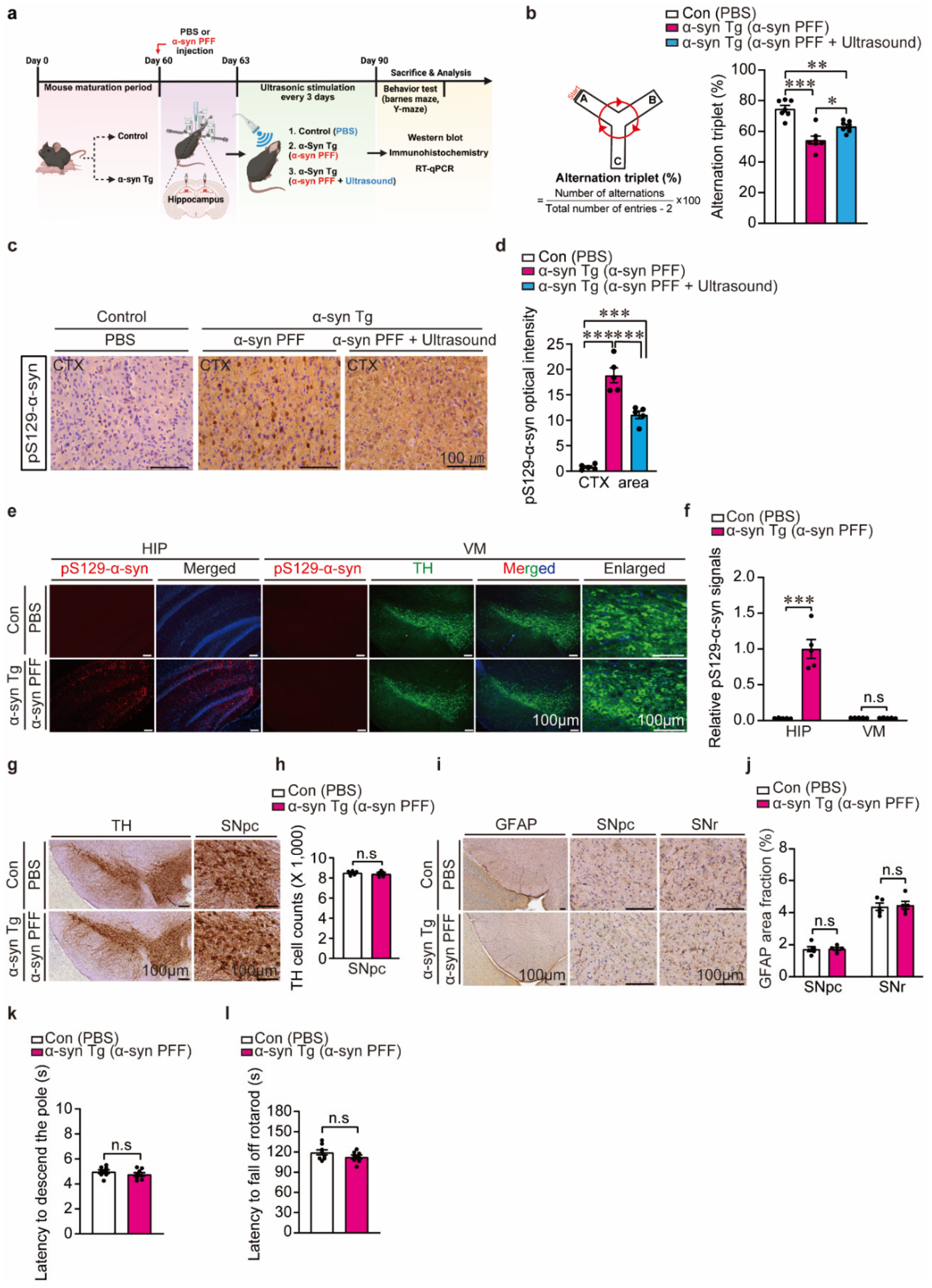

Fig. S5. Hippocampal PFF injection in α-synuclein transgenic mice induces Lewy pathology spread to the cortex, but not to the ventral midbrain, within 1 month. a Scheme of experimental procedures for α-syn PFF injection to mouse hippocampus,

transcranial ultrasound applications, behavior tests, and molecular pathway analysis *in vivo*. **b** Schematic diagram of the elevated Y-maze used to assess short-term working memory. Quantification of % alternation triplets in the indicated experimental groups (*n* = 7 mice per group). **c** Representative anti-pS129-α-syn immunohistochemistry images showing the extent of Lewy-like inclusion formation in the cortex of mice injected hippocampally with α-syn PFF with/without transcranial ultrasound applications. Scale bar = 100 µm. **d** Quantification of pS129-α-syn signal intensities in the cortex of the indicated experimental groups (*n* = 5 per group). **e** Representative immunofluorescence images showing pS129-αSyn in the hippocampus and ventral midbrain of α-synuclein transgenic mice following hippocampal αSyn PFF injection, compared with PBS-injected controls. Dopaminergic neurons in the ventral midbrain were co-labeled with anti-TH antibodies. Scale bar =100 µm. **f** Quantification of relative pS129-αSyn immunofluorescence intensities in the hippocampus and ventral midbrain across the indicated experimental groups (*n* = 5 mice per group).**g** Representative TH immunohistochemistry images of the ventral midbrain from 3-month-old α-synuclein transgenic mice with hippocampal αSyn PFF injection and PBS-injected littermate controls. Enlarged views of the substantia nigra are shown. Sections were counterstained with Nissl. Scale bar, 100 µm. **h** Quantification of TH-positive cell counts in the SNpc of the indicated groups (*n* = 5 mice per group). **i** Representative GFAP immunohistochemistry images of the ventral midbrain from 3-month-old α-synuclein transgenic mice with hippocampal αSyn PFF injection and PBS-injected littermate controls. Sections were counterstained with Nissl. Scale bar = 100 µm. **j**

Quantification of the % area fraction of GFAP signals in the indicated ventral midbrain subregions (SNpc and SNr) (*n* = 5 mice per group). **k, l** Motor performance assessed by pole and rotarod tests (n = 8 mice per group).

Data in all panels are presented as mean ± standard error of the mean. **P* < 0.05, ***P* < 0.01, and ****P* < 0.001, n.s., not significant. Unpaired two-tailed Student's t-tests or one-way analysis of variance test followed by Tukey's *post hoc* analysis. Full, uncropped original images corresponding to figure panels (S5**e**, S5**g**, and S5**i**) are provided in Fig. S8-2.

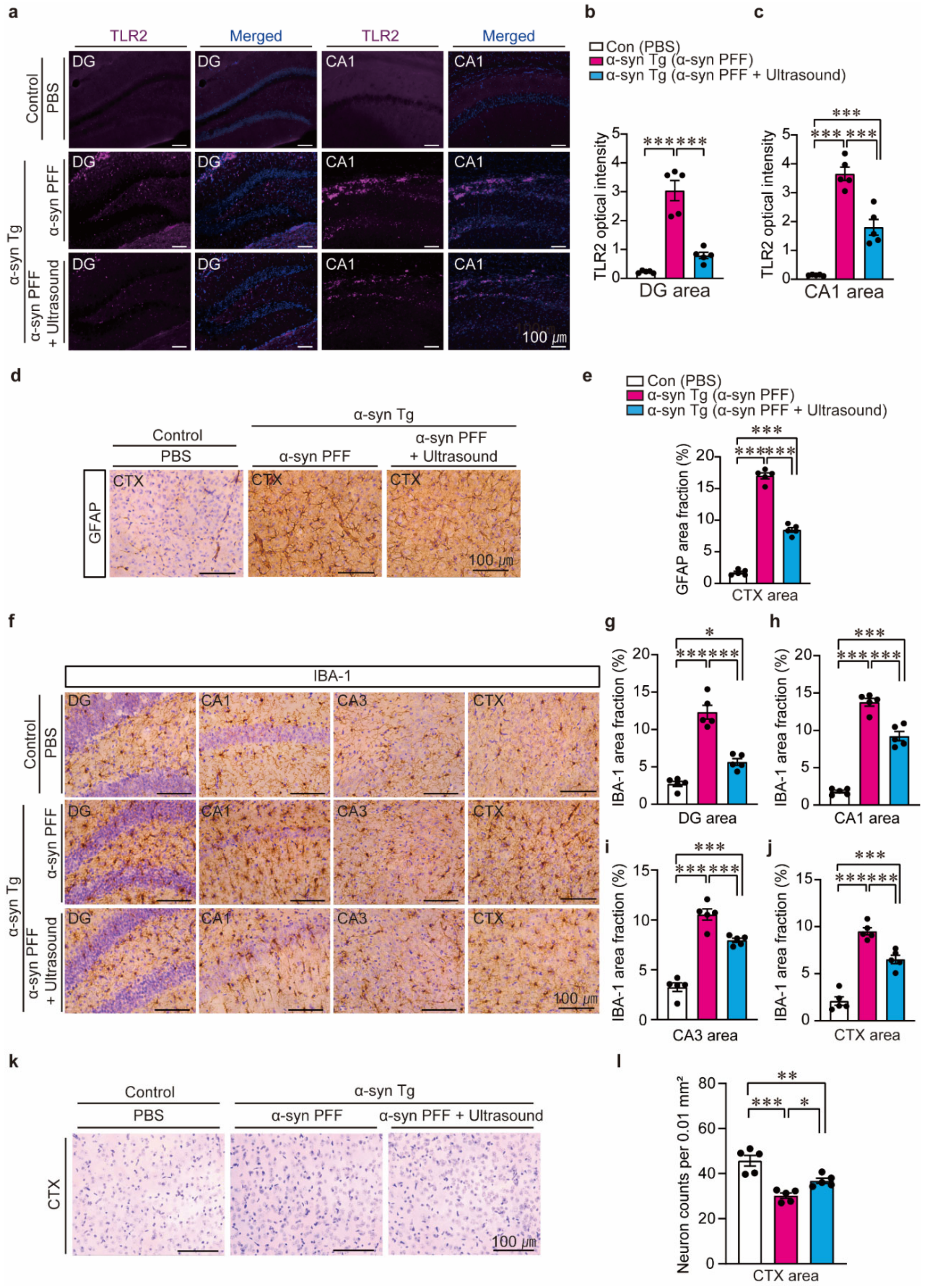

Fig. S6. Transcranial ultrasound application represses hippocampal TLR2 upregulation, microgliosis, and neurodegeneration in α-synuclein transgenic mice

with hippocampal α-syn preformed fibril (PFF) injection. a Representative anti-TLR2 immunofluorescence images of coronal hippocampal brain sections (dentate gyrus [DG] and CA1 subregions) from the indicated mouse groups. Scale bar = 100 μm. **b,c** Quantification of TLR2 immunofluorescence signal intensities in the DG and CA1 brain subregions of the experimental mouse groups (*n* = 5 mice per group). **d** Neuroinflammation in the cortex brain sections from the α-synuclein transgenic mice with hippocampal α-syn PFF injection with/without ultrasound application or phosphate-buffered saline-injected control mice monitored immunohistochemically using anti-GFAP. Scale bar = 100 μm. **e** % area fraction of GFAP immunohistochemical staining in the cortex brain sections from the indicated mouse groups (*n* = 5 mice per group). **f** Neuroinflammation in the DG, CA1, and CA3 subregions of hippocampus and cortex from the indicated mouse groups monitored using microglia marker anti-IBA-1 antibody. The brain sections were counterstained with Nissl. Scale bar =100 μm. (**g**, **h**, **i** and **j**) Quantification of IBA-1 signal intensities in the DG, CA1, and CA3 subregions of hippocampus, and cortex of the indicated mouse groups (*n* = 5 mice per group. **k** Representative Nissl-stained cortex brain sections from the indicated experimental group. Scale bar = 100 μm. **l** Quantification of Nissl-stained cortical neuron densities in the cortex sections from each mouse group (*n* = 5 mice per group). Data in all panels are presented as mean ± standard error of the mean. *$P < 0.05$, **$P < 0.01$, and ***$P < 0.001$, one-way analysis of variance test followed by Tukey's *post hoc* analysis. Full, uncropped original images corresponding to figure panels (S6**f**) are provided in Fig. S8-2.

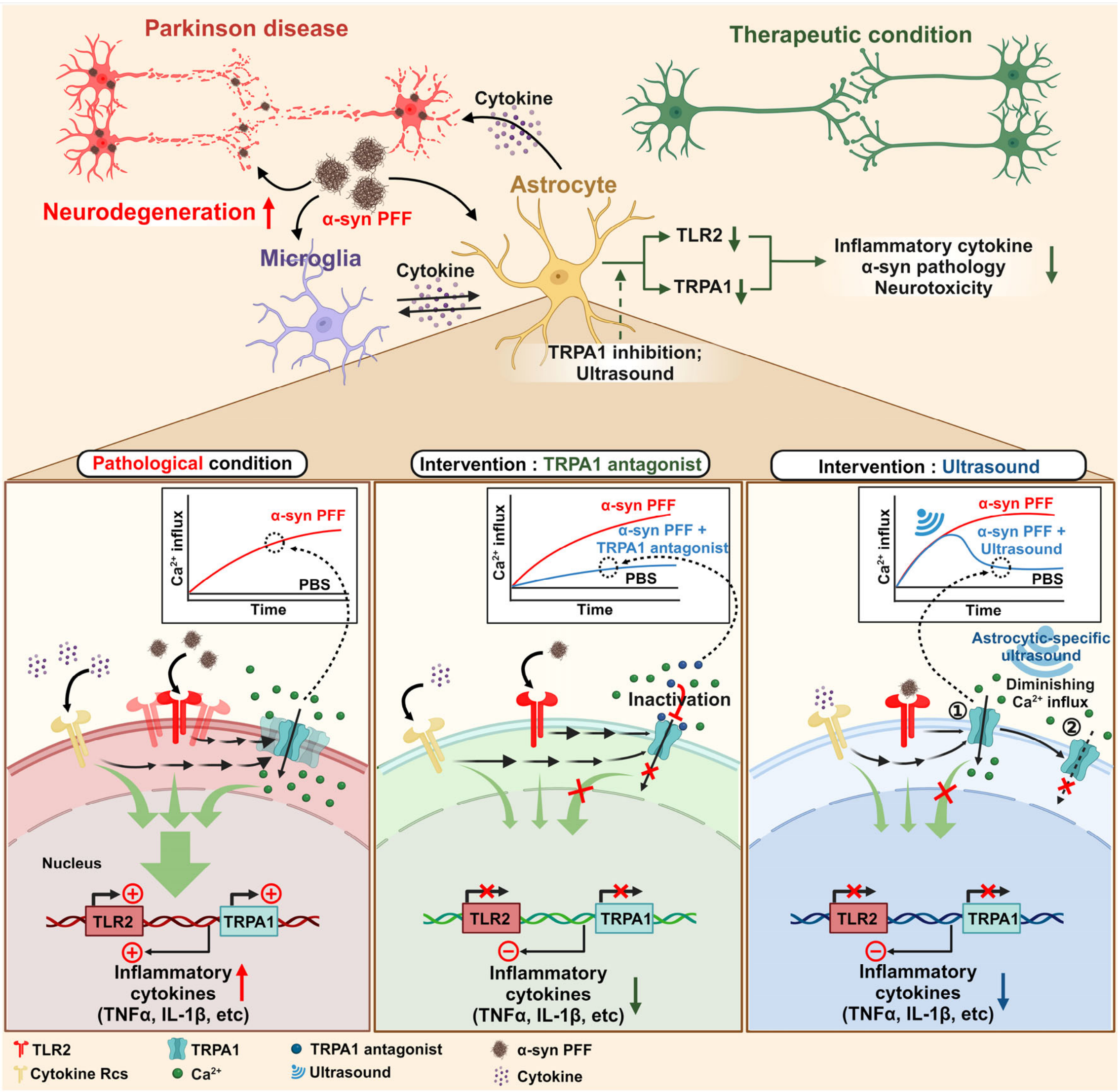

Fig. S7. Schematic illustration of ultrasound mediated astrocyte repression and neuroprotection in LBD brains. (Upper panel) α-syn PFF inoculation into the brain affects multiple cell types. Neuronal uptake of α-syn PFF induces aggregation of endogenous α-synuclein, leading to the spread of its associated pathologies. Through plasma membrane receptors such as TLR2, α-syn PFF also triggers the inflammatory activation of astrocytes and microglia. Glial proliferation and the production and secretion of inflammatory cytokines exacerbate α-syn PFF neurotoxicity in Parkinson's

disease. In this study, we report that pharmacological or ultrasound modulation of astrocyte-expressed TRPA1 blocked the α-syn PFF-induced upregulation of both TRPA1 and TLR2, preventing neuroinflammation and degeneration.

(Bottom panel) At the cellular and molecular levels, we demonstrated that α-syn PFF treatment of astrocytes results in a sustained increase in intracellular calcium, mediated by the TRPA1 ion channel. This prolonged TRPA1-mediated calcium influx subsequently led to elevated transcription of TLR2, TRPA1, and inflammatory cytokines. Low-intensity ultrasound targets mechanosensitive TRPA1 ion channels. Under pathological conditions where cytokine receptors and TLR2 are engaged by inflammatory cytokines and α-syn PFF, respectively, ultrasound stimulation suppressed TRPA1-mediated calcium influx, mimicking the anti-inflammatory effects of TRPA1 antagonists on astrocytes

Figure 4i

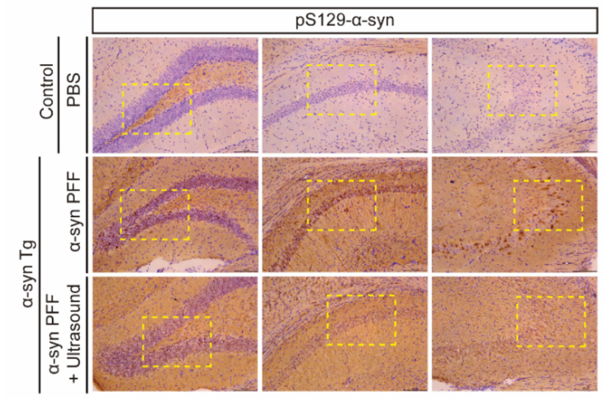

Figure 5d

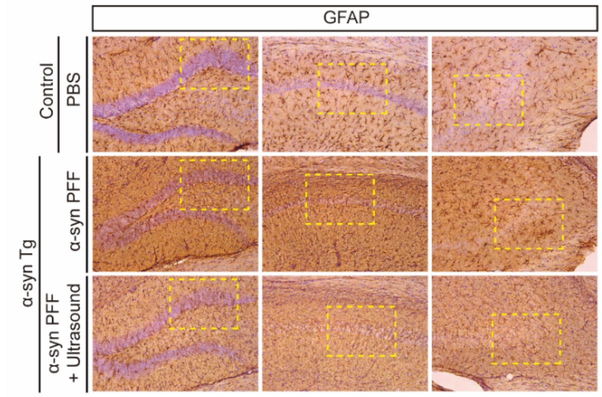

Figure 5l

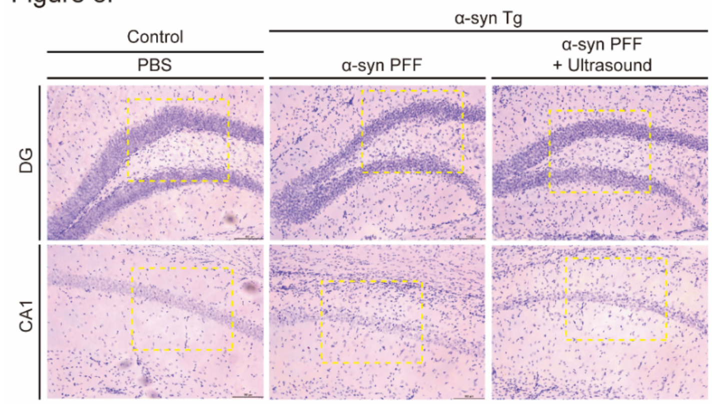

Fig. S8-1. Full, uncropped original brain section images corresponding to main and supplementary figure panels.

Full, uncropped original immunohistochemistry images acquired from the microscope

are shown for Fig. 4**i**, Fig. 5**d**, and Fig. 5**l**. Yellow dotted rectangles indicate the regions displayed in the corresponding main figure panels.

Figure S3b

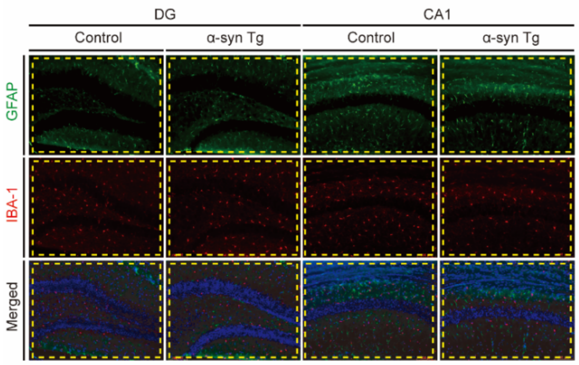

Figure S3e

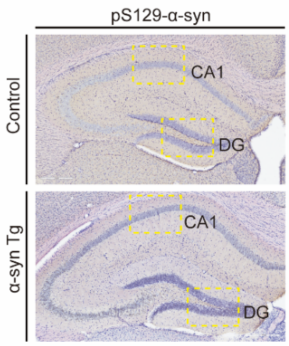

Figure S5e

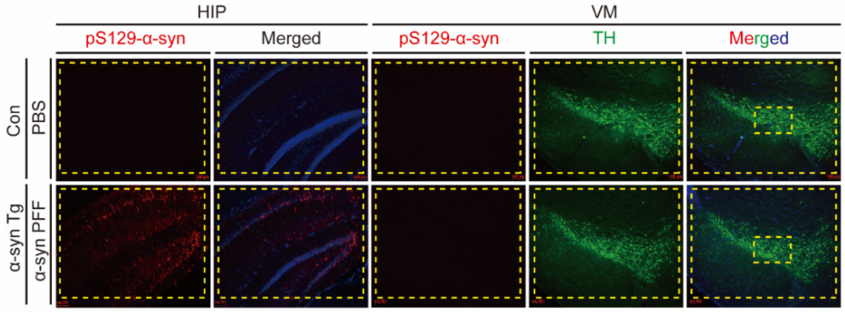

Figure S5g

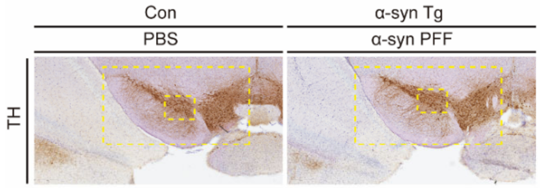

Figure S5i

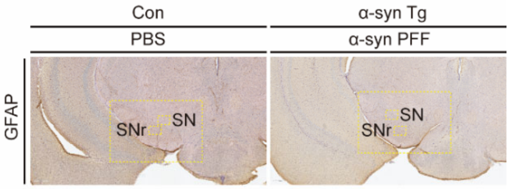

Figure S6f

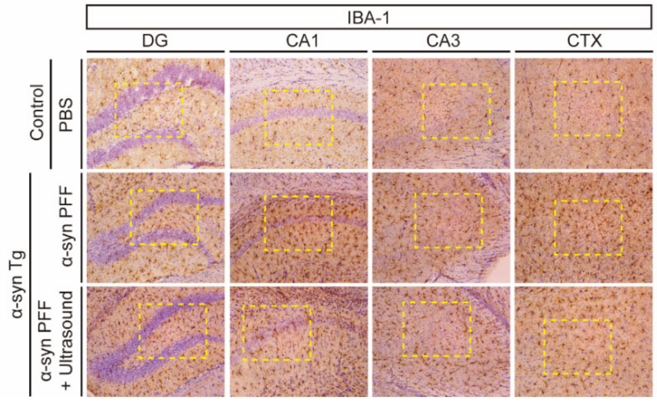

Fig. S8-2. Full, uncropped original brain section images corresponding to main and supplementary figure panels.

Full, uncropped original immunohistochemistry and immunofluorescence images acquired from the microscope are shown for Fig. S3**b**, Fig. S3**e**, Fig. S5**e**, Fig. S5**g**, Fig. S5**i**, and Fig. S6f. Yellow dotted rectangles indicate the regions displayed in the corresponding supplementary figure panels.

Supplementary Table

Supplementary Table 1. Sequence information of primers used for RT-qPCR

Gene	Forward primer	Reverse primer	Size(bp)
Tnfα	CCGATGGGTTGTACCTTGTC	TGGAAGACTCCTCCCAGGTA	217
Il-1β	GAAATGCCACCTTTTGACAGTG	TGGATGCTCTCATCAGGACAG	116
Il-1r1	CTGCTGTCGCTGGAGATTGAC	TTGGCAGGTACAAACCAAAGAT	221
Tlr2	TCTAAAGTCGATCCGCGACAT	CTACGGGCAGTGGTGAAAACT	155
Tlr4	ATGGCATGGCTTACACCACC	GAGGCCAATTTTGTCTCCACA	129
Trpa1	AACGGCTACAGCAGGGAGA	TCCAGGCACATCTTAATCATGTC	110
Gfap	AGAAAGGTTGAATCGCTGGA	CGGCGATAGTCGTTAGCTTC	299
Gapdh	AGGTCGGTGTGAACGGATTTG	TGTAGACCATGTAGTTGAGGTCA	123
