# Supplementary figures and images for: Ultrasonic repression of TRPA1-dependent astrocyte reactivity confers neuroprotection in models of Lewy body dementia
Source: Transl Neurodegener. 2026 Mar 10;15:9. doi: 10.1186/s40035-026-00544-6 (PMC12973804; doi:10.1186/s40035-026-00544-6)

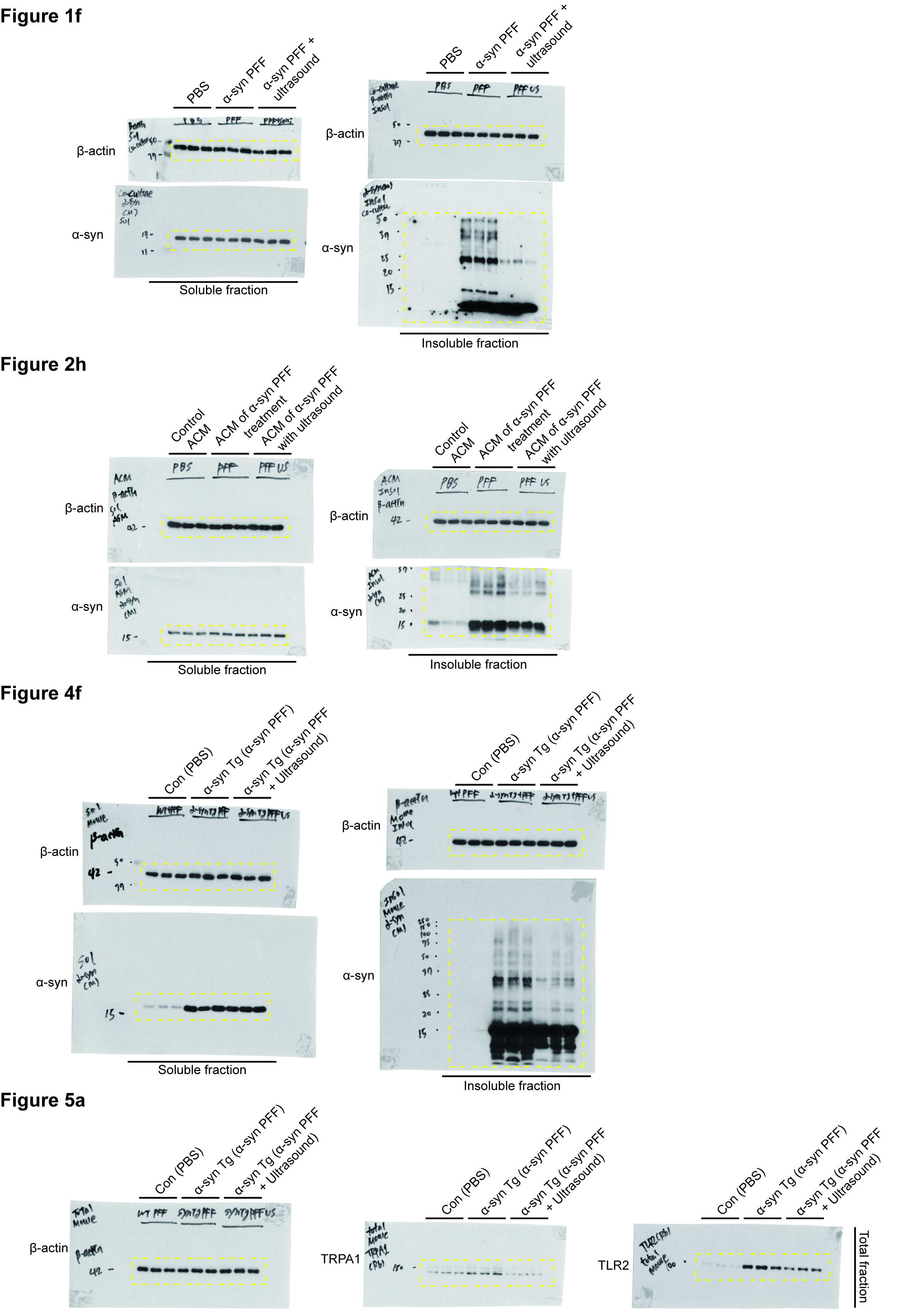

Supplement: Supplementary file 2 — Additional file 2. Full blots. [file 40035_2026_544_MOESM2_ESM.tif]
